# *Onchocerca volvulus* infection and serological prevalence, ocular onchocerciasis and parasite transmission in northern and central Togo after decades of *Simulium damnosum* s.l. vector control and mass drug administration of ivermectin

**DOI:** 10.1371/journal.pntd.0006312

**Published:** 2018-03-01

**Authors:** Kossi Komlan, Patrick S. Vossberg, Richard G. Gantin, Tchalim Solim, Francois Korbmacher, Méba Banla, Koffi Padjoudoum, Potchoziou Karabou, Carsten Köhler, Peter T. Soboslay

**Affiliations:** 1 Onchocerciasis Reference Laboratory, National Institute of Hygiene, Sokodé, Togo; 2 Institute for Tropical Medicine, University of Tübingen, University Clinics, Tübingen, Germany; 3 Centre Hospitalier Universitaire Campus, Université de Lomé, Lomé, Togo; 4 National Onchocerciasis Control Program, Kara, Togo; Emory University, UNITED STATES

## Abstract

**Background:**

Mass drug administration (MDA) of ivermectin has become the main intervention to control onchocerciasis or “river blindness”. In Togo, after many years of MDA, *Onchocerca volvulus* infection has declined dramatically, and elimination appears achievable, but in certain river basins the current situation remains unknown. We have conducted parasitological, serological, ophthalmological, and entomological assessments in northern and central Togo within the river basins of Ôti, Kéran and Mô.

**Methodology/Principal findings:**

Examinations were completed in 1,455 participants from 11 onchocerciasis sentinel villages, and *O*. *volvulus* transmission by *Simulium damnosum* sensu lato (s.l.) was evaluated. In children (aged 1–10 years), the prevalence of microfilariae (Mf) was 2.3% and in adults it ranged from 5.1 to 13.3%. Positive IgG4 responses to *O*. *volvulus* adult (crude) worm antigen (OvAg) and the recombinant Ov16 antigen were in all-ages 48.7% and 34.4%, and 29.1% and 14.9% in children, respectively. In the river basin villages of Kéran, Mô and Ôti, the IgG4 seroprevalences to OvAg in children were 51.7%, 23.5% and 12.7%, respectively, and to the Ov16 antigen 33.3% (Kéran) and 5.2% (Ôti). Onchocerciasis ocular lesions (punctate keratitis, evolving iridocyclitis and chorioretinitis) were observed in children and young adults. *O*. *volvulus*-specific DNA (Ov150) was detected by poolscreen in vector samples collected from Tchitchira/Kéran(22.8%), Bouzalo/Mô(11.3%), Baghan/Mô(2.9%) and Pancerys/Ôti(4.9%); prevalences of *O*. *volvulus* infection in *S*. *damnosum* s.l. were, respectively, 1%, 0.5%, 0.1% and 0.2%.

**Conclusions/Significance:**

In the northern and central river basins in Togo, interruption of *O*. *volvulus* transmission has not yet been attained. Patent *O*. *volvulus* infections, positive antibody responses, progressive ocular onchocerciasis were diagnosed, and parasite transmission by *S*. *damnosum* s.l. occurred close to the survey locations. Future interventions may require approaches selectively targeted to non-complying endemic populations, to the seasonality of parasite transmission and national onchocerciasis control programs should harmonize cross-border MDA as a coordinated intervention.

## Author summary

Mass drug administration (MDA) with ivermectin has become the main tool in the efforts to control and eliminate onchocerciasis (“river blindness”). In some areas, and after many years of MDA, levels of *Onchocerca volvulus* infection (the causative parasite) have declined greatly, and elimination appears achievable. In certain river basins of northern and central Togo, the present epidemiological situation remains unknown. The guidelines of the World Health Organization recommend that before ivermectin MDA can be stopped, interruption of *O*. *volvulus* transmission must be demonstrated. To this end, parasitological, serological, ophthalmological, and entomological assessments were conducted in the Ôti, Kéran and Mô river basins. *O*. *volvulus* infections and positive antibody responses were found in children aged ≤10 years and adults. Progressive ocular onchocerciasis was diagnosed, and parasite transmission by *Simulium damnosum* s.l. (the disease vector) occurred close to the survey locations. Thus, *O*. *volvulus* transmission continues in northern and central Togo, and future interventions may require approaches selectively adapted to seasonal migration of non-complying endemic populations in and out of the river basins, as well as seasonal transmission by the vectors. National control programmes should harmonize cross-border MDA as a coordinated intervention.

## Introduction

In large parts of Africa, onchocerciasis has been controlled as a public health problem by the Onchocerciasis Control Programme in West Africa (OCP) and the African Programme for Onchocerciasis Control (APOC) by mass drug administration (MDA) of ivermectin, and this intervention has been applied for more than two decades. In a vast part of the initial control areas of the OCP, *Onchocerca volvulus* infection prevalence and intensity levels have greatly declined [1, 2], and currently, the elimination of onchocerciasis appears achievable in certain endemic regions [3–7]. In Togo, the northern territories had been part of the initial OCP anti-vectorial intervention areas since 1976, whereas the central regions were included into the vector control programme in 1987, and in both areas, blackfly vector control measures were supplemented since 1988 by MDA with ivermectin. When MDA with ivermectin started, this was implemented mainly by mobile teams and the initial coverage was not very satisfactory [2]. During some years of the early 1990’s, aerial larvicide application was also suspended in several river basins. Regular epidemiological surveys conducted by the National Onchocerciasis Control Programme (NOCP) have shown that after nearly three decades of MDA in most of the onchocerciasis hyperendemic districts, the *O*. *volvulus* microfilarial prevalence has diminished below 5% in all age groups and below 1% in children aged less than 10 years, suggesting that considerable progress has been made towards the elimination of onchocerciasis according to the operational prevalence thresholds proposed in the Conceptual Framework for Elimination of Onchocerciasis by APOC [3, 8]. Parasite transmission has never been interrupted completely in central and northern Togo and Benin; the Ôti, Kéran and Mô river basins were “special intervention zones” (SIZ) where vector control and intensified ivermectin distribution needed to be continued for years after OCP closure in 2002 [9]. The interventions in the post-OCP period included continued aerial larvicide application for five additional years (2003–2007) and biannual ivermectin mass treatment was implemented until the end of 2012 [9, 10].

Despite evidence of approaching elimination in certain regions of Togo, the current situation remains to be assessed by epidemiological and entomological surveys for detection of infection in human and vector population samples according to the recent World Health Organization (WHO) guidelines [11]. The WHO guidelines suggest, firstly, that entomological evaluations by Ov-150 PCR poolscreen be conducted to demonstrate interrupted transmission of *O*. *volvulus* larvae by female blackfly vectors, and secondly, that serological evaluations by Ov-16 enzyme linked immunosorbent assay (ELISA) be carried out to determine the presence of IgG4 antibodies to the *O*. *volvulus*-specific Ov-16 antigen in children [11]. The use of skin snip microscopy in parallel with Ov-16 serology is a conditional recommendation, and it may be used in transition during the phase of monitoring and evaluation. The assessment of ocular manifestations in populations where ocular onchocerciasis was present at baseline is considered to be of low priority [11]. In the present work, parasitological, serological, ophthalmological and entomological evaluations were conducted in onchocerciasis sentinel villages in central and northern Togo to assess the current epidemiological situation and to determine whether transmission has been interrupted and ivermectin MDA can be stopped.

## Materials and methods

### Ethics statement and approval

The protocol of the study was reviewed and approved by the Togolese Bioethics Committee for Research in Health (Comité de Bioéthique pour la Recherche en Santé; CBRS, Document #013/2015/CBRS/3.Septembre 2015), and study authorization and approval were granted by the Ministry of Health of Togo (Authorization Document #338/2015/MSPS/CAB/SG). All specimens (skin snips and blood samples) used in this study were collected from study participants who provided written informed consent. The aims of the work, risks, procedures of examination and follow up were explained thoroughly to the respective village population, the village authorities and honorable community members, notably the village chief council. Consent from each study participant was documented and confirmed by signature, and consent for study participation by those younger than 18 years of age was given verbally by each participant (with written consent and approval for their participation always being obtained from their parents or accompanying responsible adults/guardians). For correct and complete understanding, explanations were always given in the local language. Before each follow-up survey, approval was obtained from the appropriate regional (Direction Régional de la Santé de la Population) and district-level (Direction Préfectural de la Santé) health authorities.

### Regular epidemiological surveys conducted by the OCP and NOCP

Regular epidemiological surveys were conducted in Togo by the OCP and the NOCP, which assessed *O*. *volvulus* microfilarial prevalence and intensity, as well as treatment coverage and compliance to ivermectin MDA within the programme area. Such surveys were performed since 1976 during the early rainy season, and around 200 participants were recruited and examined in each selected sentinel village. All sentinel villages are located within less than 3 km of distance to rivers with known breeding sites for the blackfly vector *Simulium damnosum* sensu lato (s.l.).

**Fig 1:**
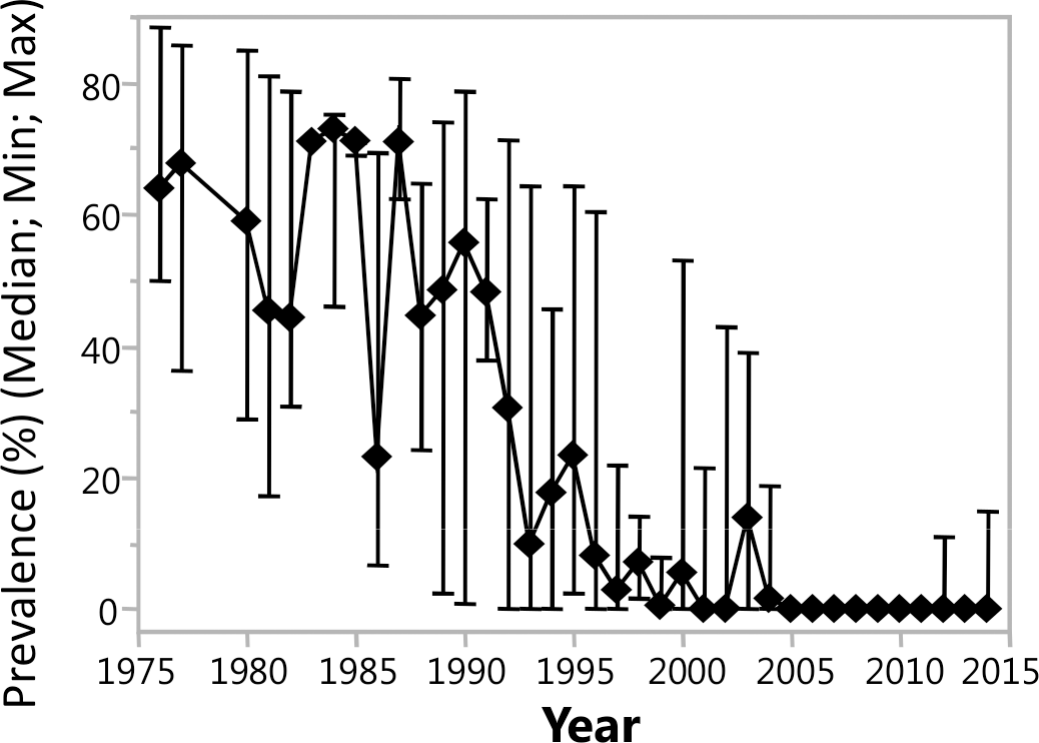
Prevalence of microfilariae (Mf) of *Onchocerca volvulus* in sentinel villages (n = 363) of the National Onchocerciasis Control Programme (NOCP) in Togo. Regular epidemiological surveys (n = 957) were conducted by the NOCP over a 37-year period (1976–2014) in onchocerciasis endemic villages, and from each participant (n = 193,742) a skin biopsy was taken from the left and right iliac crest and the emerging Mf were counted after snip incubation. The graph shows the microfilarial prevalence (median, minimum, maximum, in %) as detected during the annual surveys. Anti-vectorial interventions were applied since 1976, whereas the central regions were incorporated into the Programme in 1987. Since 1988, vector control measures were supplemented by MDA with ivermectin. Initially MDA was applied mainly by mobile teams; during some years of the early 1990’s, aerial larvicide application was suspended in several river basins. In the northern territories (SIZ) vector control and intensified ivermectin distribution was continued after OCP’s closure in 2002. Special interventions in the post-OCP period included continued aerial larvicide application for five additional years (2003–2007) and biannual ivermectin MDA until the end of 2012.

In Togo, vector control and epidemiological surveys started in 1976 within the OCP-Phase-III-Eastern Extension in the northern river basins of Ôti, Koumoungou and Kara. In 1988, control measures and epidemiological surveys began for sentinel villages of the OCP-Southern Extension in the river basins of Mô and Mono, and at the same time, also in southern Togo in the river basins of Amou, Anie and Mono. The total number of sentinel villages in Togo included in the epidemiological surveys was 363, and the endemic populations were repeatedly examined over time. Fig 1 illustrates the temporal trends in microfilarial prevalence from 1976 to 2014.

### Survey sites

**Fig 2:**
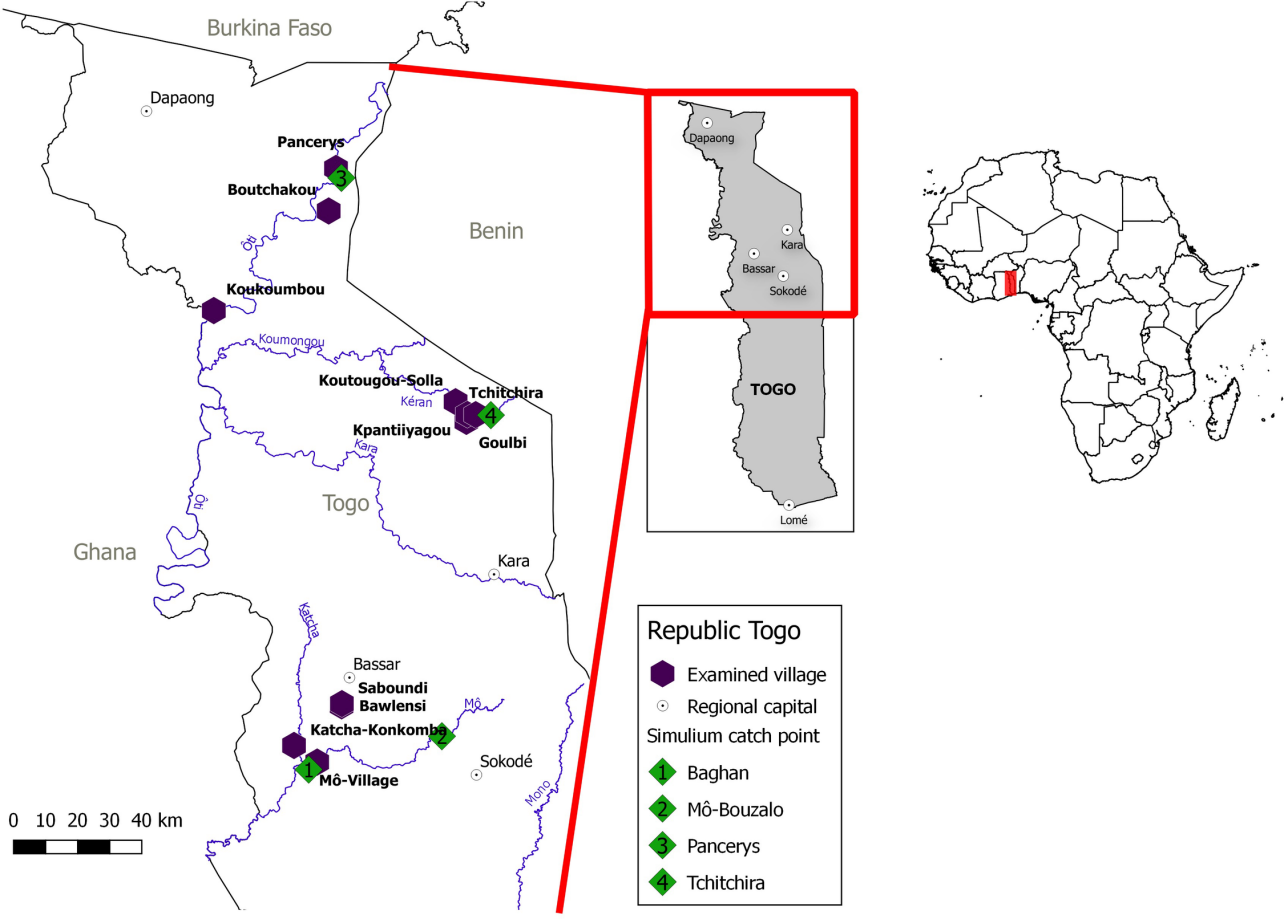
Locations of the villages surveyed and the *Simulium* collection sites in the three river basins (Ôti, Kéran, Mô) in Savanes, Kara and central regions in Togo. For an explanation of the control measures and their timings in the northern and central areas of Togo, including SIZ, see legend of Fig 1.

For this study, the parasitological, serological and ophthalmological surveys were performed in the central and northern regions of Togo (Régions Savanes and Kara), where the total populations according to the latest census, conducted in 2010, were 776,710 and 721,504 individuals, respectively. In these regions, 11 villages were selected by the NOCP for an annual survey. Fig 2 shows the selected villages and their location in Togo. Three villages are located in the Région Savanes in the Ôti river basin, i.e. Pancérys, Boutchakou and Koukoumbou. Four villages within the Kara Region are situated along the river Kara, i.e. Goulbi, Tchitchira, Koukoumbou Solla and Kpantiiyagou. Four additional villages from the Région Kara are located in the Mô river basin, i.e. Bawlesi, Mô-Village, Katcha-Konkomba and Saboundi. All sentinel villages are located within less than 1 km of distance to the rivers Ôti, Kara or Mô with known breeding sites for the blackfly vectors.

### Skin biopsy collection

Before ivermectin MDA (delivered by community-directed drug distributors, CDDs), participants gave their informed consent for the collection of skin biopsies to detect *O*. *volvulus* microfilariae (Mf). Participation and examination were conducted by family and followed the status: heads of family (parents), children, brothers, uncles, aunts, and grandparents. From each participant, a skin biopsy was taken from each the left and right iliac crest (for a total of two snips) with a sterile 2-mm Holth corneo-scleral punch biopsy tool. Immediately, skin snips were placed on glass slides and incubated with physiological saline solution for 30 minutes. Each biopsy was microscopically examined for emerging *O*. *volvulus* Mf and their number counted. After this first examination, the two biopsies were transferred separately into a round-bottom well of a 96-well plate containing saline solution, and after a 24-hour incubation biopsies were re-examined as before. The use of two incubation steps for skin biopsies is the standard procedure applied by the NOCP, and this approach makes it possible to detect *O*. *volvulus* Mf which may emerge slowly from skin. For each participant, village of residence, family affiliation, age, sex, number of ivermectin treatment rounds received and microfilarial counts in skin biopsies were recorded.

### Blood sample collection

Blood drops were collected from fingertips pricked with a sterile lancet on Whatman 903 Protein Saver Cards. The cards were air dried, sealed in plastic bags and stored at 4°C until further use. As per skin biopsies and for each participant, village of residence, family affiliation, age, sex, number of ivermectin treatment rounds received and microfilarial counts in skin biopsies were recorded.

### Ophthalmological examinations

All participants gave their informed consent for having an ocular evaluation and were examined by experienced ophthalmologists for ocular onchocerciasis lesions and additionally present ocular pathology. The ophthalmology examinations were performed by MB and TS and ocular pathologies, their grades of evolution and extent were classified as described previously [12]. All participants acknowledged having received ivermectin annually for several years through community directed treatment with ivermectin (CDTI). In the surveyed villages, therapeutic ivermectin coverage of the eligible population had been ≥80% during the past 10 years (S1 Table). Before ophthalmological examination, each participant was asked whether he or she had taken ivermectin annually for several years, and all confirmed having received treatment through CDTI. The anterior eye segment was examined by slit lamp (Haag Streit 900) after participants were asked to sit with their heads bent between their knees for at least two minutes. This position promotes the migration of Mf within the anterior chamber of the eye to be seen and counted. The examination of the posterior segment was done with an ophthalmoscope after pupil dilation with 1% tropicamide and 10% epinephrine hydrochloride. Next to individual data (age, sex, occupation, village, number of ivermectin treatments), the microfilarial load in the anterior eye segment, punctate and sclerosing keratitis and iridocyclitis were recorded. Onchocerciasis cases with punctate keratitis were grouped according to the presence of dead Mf in the cornea (DMFC) or living Mf in the anterior chamber (MFAC) and further classified as low (presence of 1–10 Mf), moderate (11–20 Mf) or high (>20 Mf). Ocular lesions of the posterior segment were coded as evolving or advanced according to the classification adopted by WHO/OCP [13]. The ocular examinations included the testing of visual acuity eye by eye with an illiterate E chart (SNELEN) placed 6 meters away from the patient’s seat, and visual acuity was graded according to WHO/OCP criteria; those with a visual acuity on one or both eyes of <1/20 (3/60 or unable to count fingers at 3 meters) were considered blind; those with impaired vision had a visual acuity on one or both eyes of <3/10 and ≥1/20 (3/60 or unable to count fingers at 3 meters), and those with good vision had a visual acuity equal to or greater than 3/10 (6/18).

### Serological tests

For the OvAg-IgG4 ELISA, an adult worm antigen extract from male and female *Onchocerca volvulus* was used [14,15]; for the Ov16-IgG4 ELISA, the recombinant *O*. *volvulus*-specific antigen Ov16 was applied to measure serological IgG4 responses. The dry blood spot (DBS) samples collected as described above were stored refrigerated at 4°C until use. For the ELISA tests, 6-mm wide circles were punched out of the DBS cards and eluted in 200μl of phosphate buffered saline (PBS) containing 0.05% Tween20 and 5% bovine serum albumin for 2 days at 4°C in deep 96-well polystyrol plates (NUNC 278605). Microtiter plates (Costar 3690, half area) were coated with OvAg (conc. 5μg/ml) or Ov16Ag (conc. 5μg/ml) in PBS pH 7.4 overnight, after which the coating antigen solutions were discarded, and the plates were blocked with PBS-Tween20 containing 5% foetal bovine serum at room temperature for 1.5 hours. Thereafter, plates were washed with PBS-Tween20 (Sigma P3563), eluted blood samples were added without dilution and the plates were incubated at 37°C for 2 hours. After using PBS-Tween20 (Sigma P3563) for washing, an anti-human IgG4 horseradish peroxidase conjugated monoclonal antibody (Thermo Fisher Scientific, no. A10654) was added (dilution 1:500) for 1.5 hours. Plates were washed as above and tetramethylbenzidine (TMB) substrate (Thermo Scientific 34021) was added. Plates were then incubated at room temperature for 15 min and the reaction was stopped with 50 μl of 0.5M sulfuric acid (ROTH, K027.1). Optical densities (ODs) were measured at 450nm with a microplate reader (EL311, BioTex Instruments).

### *Simulium damnosum* s.l. collection

The collection of *S*. *damnosum* s.l. was conducted at specific catch points at river sites by trained fly catchers in proximity to sentinel villages in the Ôti river basin (village Pancérys/ Savanes Region), Kara (village Tchitchira/Kara Region), Mô (village Baghan/Kara Region and Bouzalo/Central Region) during the rainy season on five consecutive days in late August and beginning of September 2015. Collections took place from 7am to 6pm alternating the fly catchers every two hours. The blackflies caught daily were first frozen, then suspended in 70% alcohol, and 25 individual *S*. *damnosum* s.l. were pooled into a single tube in ethanol and stored below -20°C until DNA extraction and real-time-PCR (rtPCR). In addition, repeated weekly collections of *S*. *damnosum* s.l. were continued at the river Mô site, in proximity to the village Bouzalo (Region Centrale) until August 2017. The sampling procedure was the same as above, and this long-term 2015–2017 collection was used to determine the annual biting rate (ABR) for the year 2016, which was calculated by multiplying the average number of blackflies caught daily by the number of days per week in the month to add up to 12 months.

### *O*. *volvulus*-specific Ov150-real-time-PCR

From each fly catch location, the daily pooled *S*. *damnosum* s.l. flies were processed using the Qiagen DNA Mini Kit (Qiagen, Hilden, Germany). Whole blackflies were used for rtPCR analyses. The pools were first freeze-thawed three times in liquid nitrogen, then ground with a mini grinder in a 1.5ml micro-centrifuge tube and digested with proteinase K overnight at 56°C. The eluted DNA concentration for each sample was determined by absorbance at 260 nm and DNA was stored at -20°C before PCR analysis. The DNA concentrations extracted from fly pools ranged from 540 ng/μl to 1200 ng/μl. Real-time PCR primers and probe used were as follows: OvFWD 5'-TGT GGA AAT TCA CCT AAA TAT G-3', OvREV 5'-AAT AAC TGA CCT ATG ACC-3', OvProbe 5'-FAM-TAG GAC CCA ATT CGA ATG TAT GTA CCC-TAM-3' (Eurofins, Genomics). Primers and TaqMan probe sequences were designed to amplify a fragment of *O*. *volvulus* repeat DNA (Ov-150 bp, GenBank accession number: J04659.1). Taqman Universal PCR Master Mix (Applied Biosystems, P/N 4304437) and nuclease-free water were used with all reactions with the following concentrations and volumes: 2.5 μl of 20 μM OvFWD, 2.5 μl of 20 μM OvREV primer, 1.5 μl of 9.2 μM OvProbe, 27.5 μl of 2×Master Mix, 50 ng of template DNA from extracted *S*. *damnosum* s.l. pools, or 1 ng of genomic DNA isolated from adult *O*. *volvulus*, and nuclease-free water was added up to a final volume of 55 μl. Reactions (2 × 25 μl per well) were run with the following cycling conditions: 50°C for 2 min, 95°C for 10 min, (95°C for 15 s, 49°C for 30 s, 60°C for 2 min) × 40 cycles. The Applied Biosystems 7300 Real Time PCR System (96-well format) SDS version 1.4 software was used for *S*. *damnosum* s.l. pools collection in 2015 and for blackfly pools from 2016 the Corbett Rotor Gene RG-300, version 6, software was applied. Duplicate blackfly pool DNA samples with a cycle threshold (Ct) value of less than 30 were considered to be positive for *O*. *volvulus* DNA.

### Data analysis

Data were entered in Microsoft Excel and analyses were conducted with the statistical software SAS JMP 11.1.1. For Mf prevalence values, the 95% confidence intervals (95% CI, Wilson score interval) were calculated. The sensitivity of the *O*. *volvulus*-specific IgG4 ELISA was determined with a contingency analysis. For explorative data analyses, the two-sample Wilcoxon test was applied to evaluate differences between groups. The Chi-square test was used to test differences between examined males and females (e.g. participation rates). Fisher’s exact test (two-sided) was applied to compare Mf-prevalence and the ELISA IgG4-OvAg and Ov-16 positive responses between the river basins (Kéran, Ôti, Mô), and the number of Ov-150 DNA positive *Simulium damnosum* s.l. pools from Ôti/Pancery, Kéran/Baghan, Kéran/Tchitichira and Mô/Bouzalo. One-sided Fisher's exact test was used to evaluate differences in the prevalence of ocular pathologies in patients from the Ôti, Kéran and Mô river basins. Correlations between ophthalmological variables as well as between these and age were explored with Spearman correlation coefficient. For multiple testing, the application of the Bonferroni Holm adjustment (11 villages, 3 river basins, 7 age groups, microfilarial prevalences, IgG4 responses) resulted in an alpha level of 0.0023. For multiple comparisons, and to avoid type I errors, differences between groups were analyzed by the Tukey-Kramer Test.

## Results

### *Onchocerca volvulus* microfilarial prevalence in OCP sentinel villages during the past decades

The onchocerciasis control measures continuously applied from 1976 until 2002 consisted of aerial application of larvicidal compounds into the simuliid vector breeding sites, and from 1990 onwards, annual MDA of ivermectin was introduced, continuing until the present. In 1976, in most locations the prevalence of *O*. *volvulus* infection exceeded 50%, and 20 years later, Mf positivity in the survey populations decreased to below 20% (median) (Fig 1). The *O*. *volvulus* microfilarial prevalence in onchocerciasis sentinel villages located in the major river basins of Ôti, Kéran, Kara, Mô, Koumoungou, Anie and Mono declined markedly (Fig 1), and until the year 2014, the median prevalence of *O*. *volvulus* infections dropped below 5%, but in several locations the Mf-positivity exceeded this level in the river basins of Ôti, Kéran and Mô.

### Participants’ characteristics

In 2015, a total of 1,455 individuals from 11 NOCP sentinel villages gave their informed consent for participation. Of these, the proportions (and numbers) of individuals originating from each river basin were: 22.3% (n = 324) in Ôti; 37.0% (n = 539) in Kéran, and 40.7% (n = 592) in Mô. Table 1 summarizes the numbers examined by age and sex and the proportion of positives for skin Mf. Information on age is missing from 41 of the 1,455 participants, so data in Table 1 are reported for a total 1,414 individuals. Of these, 819 were females and 595 males. The median age in females and males was 30 and 29 years, respectively. Until the age of 15 years, girls and boys were similarly represented in the survey, but participation in examination (and treatment) of men aged 16 to 40 years decreased significantly (Table 1). The Chi-square test was applied to compare differences between female and male survey participation within age groups, indicating a statistically significant difference with greater participation of females (16-20y: p = 0.0007; age groups 21-25y, 26-30y and 31-35y: for each p<0.0001; 36-40y: p = 0.04). In the age groups above 40 years, differences in participation between the sexes were not significant (Table 1).

**Table 1:** *Onchocerca volvulus* microfilarial prevalence (% Mf-positive) by age and sex groups in the survey participants from NOCP sentinel villages in central and northern Togo. The number of examined participants, the Mf-positive status and the percentage of Mf-positive individuals are shown. From n = 41 study participants the age is missing. The 95% confidence intervals (95% CI, Wilson score interval) of the prevalence values are indicated in square brackets. (* significant differences between female and male survey participation).

Age group	Examined (female/male)	% in population	Mf-positive (female/male)	% Mf-positive [95% CI]
5-10y	172 (76/96)	12.2	4(3/1)	2.3 [0;5.8]
11-15y	212 (106/106)	15.0	4(3/1)	1.9 [0;5.0]
16-20y	95 (64/31)*	6.7	1(1/0)	1.1 [0;5.7]
21-25y	118 (86/32)*	8.3	6(4/2)	5.1 [0.8;9.3]
26-30y	195 (137/58)*	13.8	16(7/9)	8.2 [4.9;11.5]
31-35y	135 (90/45)*	9.5	13(7/6)	9.6 [5.7;13.6]
36-40y	105 (63/42)*	7.4	14(8/6)	13.3 [8.8;17.8]
41-45y	89 (44/45)	6.4	7(3/4)	7.9 [3.0;12.7]
46-50y	90 (53/37)	6.4	6(3/3)	6.7 [1.8;11.5]
51-55y	62 (30/32)	4.4	3(0/2)	3.2 [0;9.0]
56-60y	61 (32/29)	4.3	4(3/1)	6.6 [0.7;12.4]
61-80y	80 (38/42)	5.6	6(4/2)	7.5 [1.6;13.4]
	1,414 (819/595)*	100	83 (46/37)	5.9 [4.6;7.1]

### *Onchocerca volvulus* microfilarial prevalence

A total of 1,455 individuals were examined by skin biopsy for *O*. *volvulus* Mf with 83 positives; the overall Mf prevalence in the survey participants was 5.7% [4.5;6.9] (Table 2). In the Ôti, Kéran and Mô river basins, the ranges of Mf prevalence were 0.8–5.3%, 7.7–13.6% and 0–8.6%, respectively. The districts in northern and central Togo, where the surveyed 11 villages are located, formed part of the SIZ, and here MDA attained ≥80% (median) treatment coverage of the eligible population from 2005 until 2015 (S1 Table).

**Table 2:** The *Onchocerca volvulus* microfilarial (Mf) prevalence in all age participants, the IgG4 positive responses specific for *Onchocerca volvulus* adult worm antigens (OvAg) and for the *O*. *volvulus*-specific antigen Ov16 in all-ages participants and in children ≤10 years in NOCP sentinels villages in central and northern Togo by river basins. The total number of participants and of children ≤10 years examined and their positive IgG4 responses to OvAg and Ov16 are indicated. Numbers in square brackets indicate the 95% confidence intervals.

		*Onchocerca volvulus* infection			IgG4-OvAg ELISA						IgG4-Ov16 ELISA					
River Basin	Village	examined all ages (n)	Mf-positive (n)	% Mf positive	positive (n)	% all ages positive	children (5-10y)	children positive	% children positive		examined all ages (n)	positive (n)	% all ages positive	children (5-10y)	children positive	% children positive
Ôti	Pancérys	140	4	2.9 [0;6.6]	34	24.3 [17.1;31.4]	32	3	9.4 [0;24.2]		92	10	10.9 [4.4;17.4]	25	1	4.0 [0;12.3]
Ôti	Boutchakou	127	1	0.8 [0;4.8]	32	25.2 [17.5;32.6]	17	3	17.6 [0;39.9]		92	15	16.3 [8.6;24.0]	15	1	6.7 [0;21.0]
Ôti	Koukoumbou	57	3	5.3 [0;11.2]	5	8.8 [1.2;16.3]	14	2	14.3 [0;36.6]		70	9	12.9 [4.8;20.9]	17	1	5.9 [0;18.4]
**Ôti River Basin**		**324**	**8**	**2.5 [0;4.9]**	71	21.9*** [17.4;26.4]	63	8	12.7^&&^ [2.1;23.3]		254	34	13.4^&&^ [9.2;17.6]	57	3	5.3^&&^ [0;11.2]
Kéran	Goulbi	131	15	11.5 [7.5;15.4]	89	67.9 [59.8;76.0]	6	3	50 [15.8;84.1]		92	42	45.7 [35.3;56.0]	1	0	0
Kéran	Tchitchira	146	15	10.3 [6.6;14.0]	101	69.2 [61.6;76.7]	4	2	50 [8.2;91.8]		92	42	45.7 [35.3;56.0]	1	0	0
Kéran	Koutougou Solla	81	11	13.6 [8.6;18.5]	53	65.4 [54.9;76.0]	9	7	77.8 [49.9;105.6]		79	45	56.0 [45.8;68.1]	6	4	66.7 [12.4;120.6]
Kéran	Kpantiiyagou	181	14	7.7 [4.4;11.1]	116	64.1 [57.0;71.1]	39	18	46.2 [32.7;59.5]		92	46	50.0 [39.6;60.4]	22	6	27.3 [7.1;47.5]
Kéran River Basin		539	55***	10.2*** **[8.3;12.1]**	359	66.6*** [62.6;70.6]	58	30	51.7^&^ [40.7;62.8]		355	175	49.3 [44.1;54.5]	30	10	33.3 [15.4;51.2]
Mô	Bawlensi	148	0	0 [0;3.7]	46	31.1 [23.5;38.6]	11	2	18.2 [0;43.4]		n.d.			n.d.		
Mô	Mô-Village	151	13*	8.6 [4.9;12.2]	91	60.3 [52.4;68.2]	11	4	36.4 [11.1;61.6]		22	9	40. 9 [18.6;63.2]	n.d.		
Mô	Katcha-Konkomba	188	5	2.7 [0;5.9]	74	39.4 [32.3;46.4]	20	2	10.0 [0;28.7]		n.d.			n.d.		
Mô	Saboundi	105	2	1.9 [0;6.3]	67	63.8 [54.5;73.2]	9	4	44.4 [16.6.;72.3]		13	1	7.7 [0;24.4]	n.d.		
Mô River Basin		592	**20**	**3.4 [1.5;5.2]**	278	47.0*** [42.9;51.0]	51	12	23.5 [11.7;35.3]		35	10	28.6 [12.8;44.3]	n.d.		
**TOTAL**		**1455**	**83**	**5.7 [4.5;6.9]**	**708**	**48.7 [46.1;51.2]**	**172**	**50**	**29.1 [22.;35.9]**	** **	**644**	**219**	**34.0 [30.3;37.7]**	**87**	**13**	**14.9 [7.3;22.6]**

*** Fisher’s exact Test (two-sided) with p<0.0001 compared to the other river basins

^&&^ Fisher’s exact Test (two-sided) with p<0.001 compared to the river basin of Kéran

^&^ Fisher’s exact Test (two-sided) with p<0.003 for Kéran river basin compared to the river basin of Mô; n.d.: not done

### IgG4 responses to *O*. *volvulus* (crude) adult worm antigen (OvAg) and to Ov16 antigen

**Fig 3:**
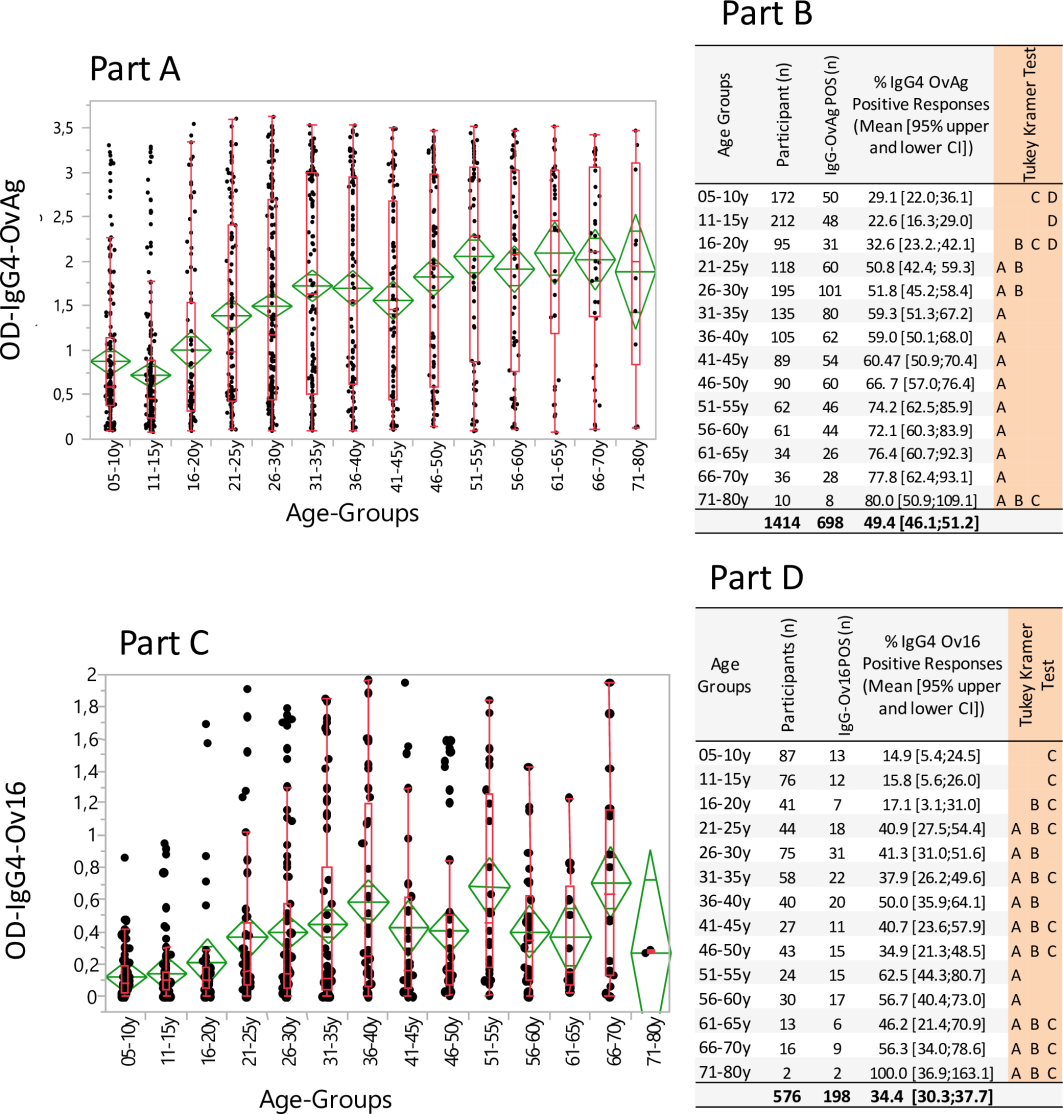
*Onchocerca volvulus* adult worm (OvAg) and recombinant Ov16 antigen-specific IgG4 reactivity (optical densities; OD) in participants and positive and negative IgG4 responses in age groups. In A and C the data on antigen-specific IgG4 reactivity are shown as mean optical densities (ODs) with 95% confidence intervals for the means (diamonds). The data presented in box plots show the median OD per age group with the 25% and 75% quartiles and the 1.5x of the interquartile range. In B and D the antigen-specific-IgG4 positive and negative responses in age groups are indicated (in %).

The sensitivities of the OvAg- and Ov16-specific IgG4-ELISAs to detect Mf-positive participants were, respectively, 89.2% and 71.4% (Table 3). The all-ages IgG4 seroprevalence values for OvAg and Ov16 were, respectively, 49.4% and 34.4% (Table 2). Age-specific serological IgG4 responses to the OvAg and Ov16 antigens are shown in Fig 3. OvAg and Ov16 sero-prevalence increases with age (OvAg: Spearman ρ = 0.346, Ov16: Spearman ρ = 0.299, both p<0.0001) (Fig 3) and it attains a maximum level around the age of 50 years. During the first two decades of age, the mean OvAg- and Ov16-specific IgG4 reactivity was low in most cases; from 16 years onwards an enhanced responsiveness was observed, and from 20 years and older the mean participants' serologic IgG4 responses to OvAg and Ov16 continued to rise steadily until the fifth decade of age (Fig 3). In children of 5–10 years, 29.1% and 14.9% showed a positive IgG4 serological response to OvAg and Ov16 (Fig 3). In the Kéran, Mô and Ôti river basins, IgG4 sero-prevalence values in children (5–10 years) to OvAg were 51.7%, 23.5% and 12.7%, respectively, and to Ov16 the values were 33.3% in Kéran and 5.2% in Ôti (Table 2).

**Table 3:** The sensitivity of the IgG4 ELISAs based on *Onchocerca volvulus* adult worm antigen (OvAg) (left panel) and on the *O*. *volvulus*-specific recombinant antigen Ov16 (right panel) for the detection of patent *O*. *volvulus* infection (Mf positivity). The contingency table indicates, in the upper left and right panels, the percentage of false-negative results in relation to Mf positive test results. In the lower left and right panels, the percentage of correct positive results in relation to Mf positivity is highlighted for the OvAg-IgG4 and Ov16-IgG4 ELISAs. The cutoff for IgG4-OvAg and IgG4-Ov16 positive responses was set at the upper limit of the 95% confidence interval of the mean optical density (OD) in *O*. *volvulus* microfilariae (Mf) negative 5–10 year old children.

OvAg-IgG4					Ov16-IgG4			
	Ov-Mf-neg	Ov-Mf-pos				Ov-Mf-neg	Ov-Mf-pos	
OvAg-IgG4-neg	738	9	747		Ov16-IgG4- NEG	403	22	425
	53.8	**10.8**				71.1	**28.6**	
OvAg-IgG4-pos	634	74	708		Ov16-IgG4- POS	164	55	219
	46.2	**89.2**				28.9	**71.4**	
	1372	83	1455	TOTAL		567	77	644
	94.3	5.7		%		88.0	12.0	

### *O*. *volvulus*-specific DNA (Ov150) in *Simulium* spp. black flies collected

**Fig 4:**
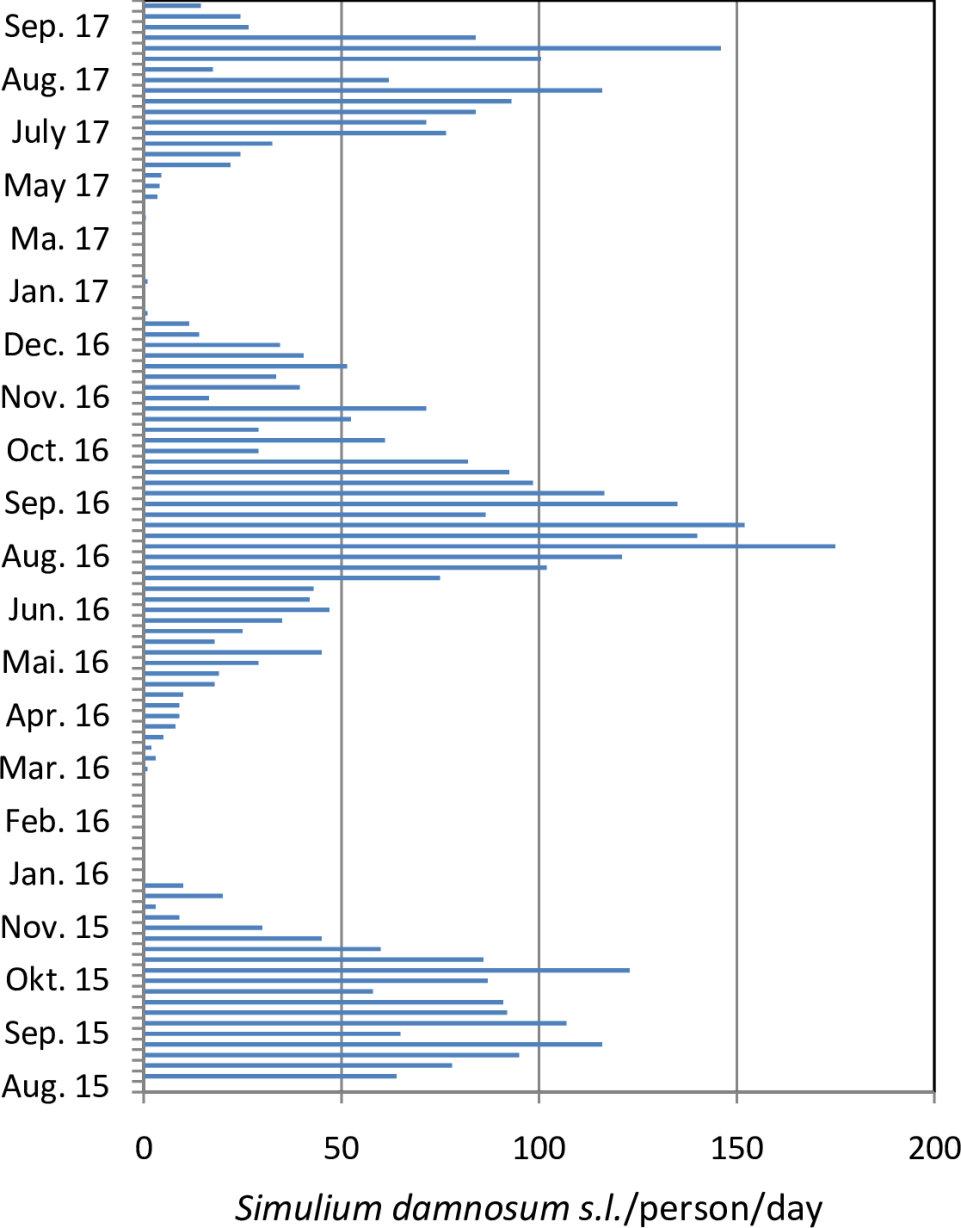
Collection of *Simulium damnosum* s.l. at the catch point at the Mô river (village of Bouzalo) caught per person (one day per week). The annual biting rate (ABR) from January 2016 until December 2016 was calculated by multiplying the number of blackflies caught daily by the number of days per week for each month to add up to 12 months. Collections were conducted from 7am to 6pm with alternating fly catchers every two hours as described in Material and Methods.

In total, 5,575 blackflies were grouped in 223 pools (25 flies each). Eight out of 35 pools from Tchitchira were positive for Ov150-DNA (22.8%) and the calculated prevalence of *O*. *volvulus* infection in *S*. *damnosum* s.l. was 1% [16]. In Baghan, three positive pools were detected (2.9%) and the calculated infecton prevalence was 0.1%. In Mô, five positive *S*. *damnosum* s.l. pools were identified (11.3%) with a 0.5% prevalence of *O*. *volvulus* in blackflies (Table 4). At Pancéry, in the Ôti river basin, two *O*. *volvulus*-positive pools (4.9%) were found, with a 0.2% prevalence of *O*. *volvulus* in black flies. *O*. *volvulus*-DNA-positive pools from Mô were found from early August 2015 until late October 2015, suggesting that transmission of *O*. *volvulus* occurred during the rainy season. An annual biting rate (ABR, Jan 2016-Dec 2016) was calculated as 15,519 bites/person/year at Mô-Bouzalo (Fig 4).

**Table 4:** Ov150 rt-PCR (Poolscreen) testing of *Simulium damnosum* s.l. black flies collected at sentinel catch points in northern and central Togo in 2015. Each pool consists of 25 blackflies and is listed with the total number of tested flies by catch point, and the number of Ov150-positive pools. The prevalence (in % with 95% confidence intervals) of *O*. *volvulus* in *S*. *damnosum* s.l. is calculated according to Katholi et al. 1995 [16]. Fisher’s exact test was applied to evaluate differences in infection rates between pools.

Ov150 rt-PCR		
Locality (no. flies tested)	Ov-150 positive pools	Prevalence of *O*. *volvulus* in *Simulium damnosum* s.l.
Ôti/Pancery (41x25 = 1,025)	2	0.2% [0.03; 1.3]
Kéran/Tchitchira (35x25 = 875)	8*	1.0% [0.9; 2.1]
Mô/Baghan (103x25 = 2,575)	3	0.1% [0.03; 0.5]
Mô/Bouzalo (44x25 = 1,100)	5	0.5% [0.2; 1.3]

p<0.05, difference between Ôti/Pancery, Kéran/Baghan and Mô/Bouzalo (Fisher’s exact test).

### Ocular pathology

**Fig 5:**
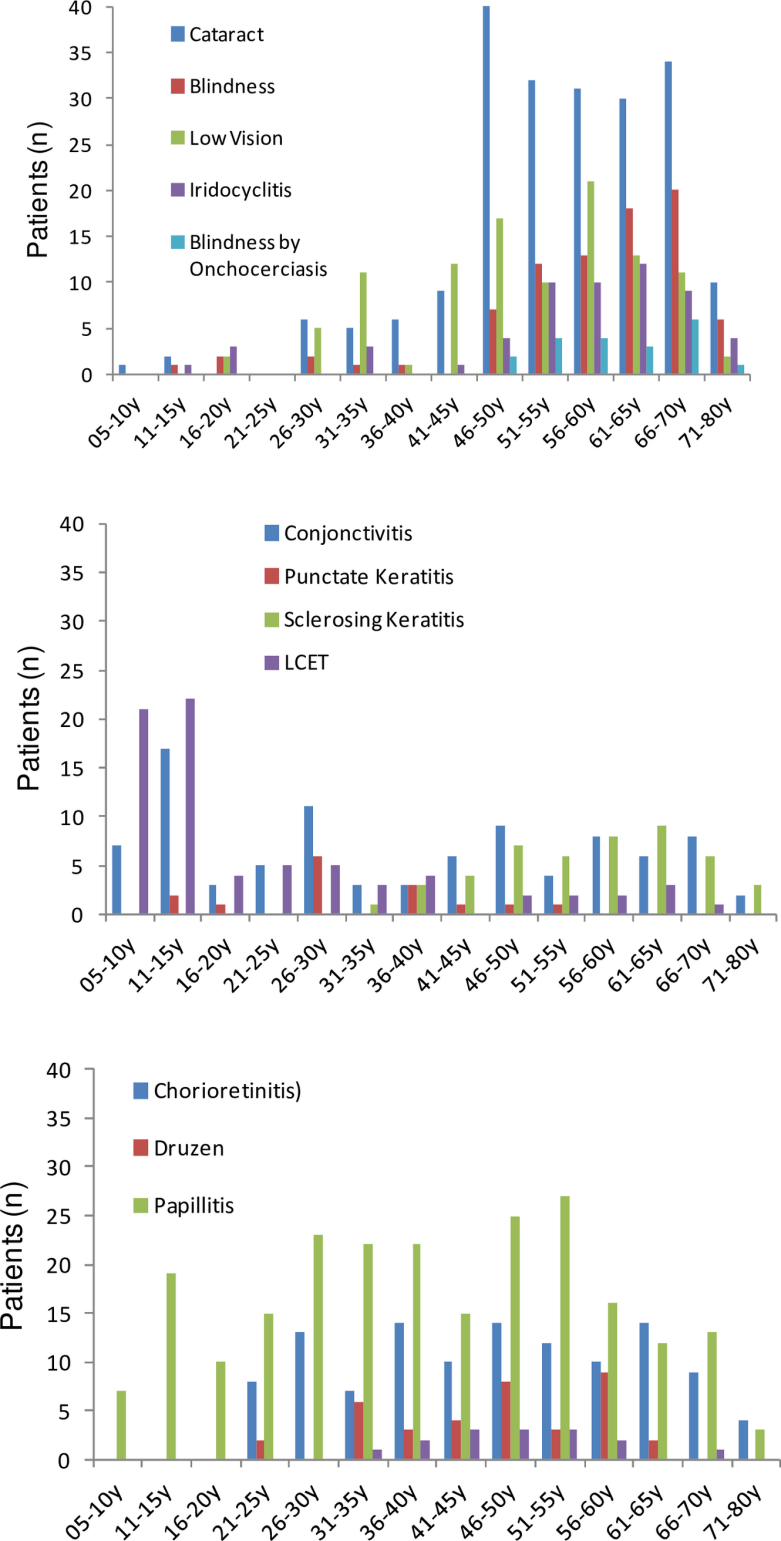
Ocular pathologies and visual acuity in study participants (n = 1,172) according to age. The ocular pathologies, their grades of evolution and extent were classified as described previously [12].

The main ocular pathologies in the examined village populations, reported for the right eye, were papillitis (19.5%), cataract (17.6%), chorioretinitis (9.8%), conjunctivitis (7.8%), tropical limbo-cojunctivitis (LCET) (6.3%), iridocyclitis (4.6%), sclerosing keratitis (3.9%) and blindness of either eye (7.4%) (Table 5). Of note were punctate keratitis lesions with 1–10 Mf of *O*. *volvulus* in the cornea present in children (aged 12–15 years) and adults (28–52 years) and sclerosing keratitis in adults (Table 5, Fig 5). In four cases, alive or dead *O*. *volvulus* Mf were detected in the anterior chamber of the eye. Iridocyclitis in evolution (n = 7) was found in youngsters and adults; retinal lesions (chorioretinis) were present in younger adults and older ages (Fig 5) with n = 19 being in evolution and n = 41 at an advanced stage. Evolving cataract (all causes) was diagnosed in a few children and mainly in older ages. Cataracts caused by *O*. *volvulus* infection and blindness caused by onchocerciasis were observed in individuals aged above 50 years (Fig 5). Cataract, blindness, chorioretinitis, iridocyclitis, punctate and sclerosing keratitis were observed similarly in female and male participants, while LCET, papillitis, trichiasis and low vision were more often diagnosed in females (Table 5). Cataract (Spearman's rank correlation: ρ = 0.71; p = 0.005), blindness (ρ = 0.75; p = 0.002), sclerosing keratitis (ρ = 0.78; p = 0.001), iridocyclitis (ρ = 0.71; p = 0.005) and low visual acuity (ρ = 0.56; p = 0.04) correlated positively with age disclosing the age-related decline in visual functions (Table 5). LCET and normal vision were negatively correlated with participants’ age (Table 5). Several manifestations (right eye), notably cataract, sclerosing keratitis and iridocyclitis, were diagnosed less often (p<0.001, one-sided Fisher exact Test) in patients from villages situated in the Mô river basin (Table 5). Of note were the positive correlations of cataract with sclerosing keratitis (Spearman's rank correlation: ρ = 0.874; p<0.0001), cataract with iridocyclitis (ρ = 0.716; p = 0.0004), with blindness (ρ = 0.765; p = 0.0014) and with low visual acuity (ρ = 0.753; p = 0.0019) (Table 6). Strongly associated (ρ = 0.8134, p = 0.0004) were sclerosing keratitis with iridocyclitis, both pathologies of the anterior segment of the eye, and similarly, vascular retinopathy and druzen correlated positively (ρ = 0.7723, p = 0.0012) (Table 6), both are posterior eye segment lesions.

**Table 5:** Ocular pathologies in study participants (n = 1,183) from onchocerciasis endemic villages situated in the river basins of Kéran, Mô and Ôti in northern and central Togo. The village populations have been treated annually via CDTI. The ocular pathologies, their grades of evolution and extent were classified as described previously [12] and are indicated for the right and left eye (RLE), the left eye (LE) and the right eye (RE). Chi-Square tests were applied to compare ocular pathologies of the right eye (RE) between female and male patients. One-sided Fisher exact test was used to evaluate differences in the prevalence of ocular pathologies in patients from the Ôti, Kéran and Mô river basins, and significant differences are indicated by p<0.05. Spearman’s rank correlation analyses of ocular pathologies of the right eye (RE) and age were conducted; the correlation coefficient ρ and the significant associations (p<0.05) are shown.

Ocular Pathology	RLE	RE	LE	% RE	% LE	RE pathology in female/male (p-value)	RE pathology in correlation (ρ) with age (p-value)	RE Pathology in Kéran(K), Mô(M), Ôti(O)]^&^ (p-value)
**Cataract**	405	206	199	17.6	16.9	n.s	ρ = 0.7087; p = 0.0045	K>M: p = 0.0009; K>O: p = 0.015; [K:111;M:84;O:11]
**Blindness**	168	84	84	7.2	7.2	n.s.	ρ = 0.7488; p = 0.0021	n.s.; [37;43;4]
**Chorioretinitis**	232	115	117	9.8	9.9	n.s.	n.s.	K>O: p = 0.014; M>O: p<0.0015; [45;68;2]
**Conjunctivitis**	183	92	91	7.8	7.7	n.s.	n.s	M>K: p = 0.03; O>K: p = 0.03; [29;52;11]
**Druzen**	78	37	41	3.1	3.4	p = 0.03; (25/12)	n.s.	K>M: p<0.007; K>O: p = 0.01; [25;12;0]
**Iridocyclitis**	112	57	55	4.8	4.6	n.s.	ρ = 0.7298; p = 0.0045	K>M: p<0.0003; [38;16;3]
**Keratitis (not onchocerciasis)**	28	14	14	1.1	1.1	n.s.	n.s.	n.s.
**Punctate keratitis**	26	15	11	1.3	0.9	n.s.	n.s.	n.s.
**Sclerosing keratitis**	93	47	46	4.0	3.9	n.s.	ρ = 0.7806; p = 0.001	K>M: p<0.0001; [34;10;3]
LCET*	148	74	74	6.3	6.3	p = 0.02; (27/47)	ρ = -0.7168; p = 0.0039	n.s.
**Papillitis**	454	229	225	19.5	19.1	p = 0.04 (130/99)	n.s.	O>K: p = 0.03; O>M: p<0.09; [99;103;27]
**Vascular retinopathy**	30	15	15	1.2	1.2	n.s.	n.s.	n.s.
**Trichiasis**	26	12	13	1.0	1.1	p = 0.004; (11/1)	n.s.	n.s.
Low vision (code WHO)**	193	105	88	4.7	4.0	p<0,000; (78/27)	ρ = 0.5583; p = 0.038	n.s.
**Normal vision (code WHO)**	2028	1011	1017	45.5	45.8	p = 0.0062; (548/461)	ρ = -0.8263; p = 0.0003	n.s.

RLE = Right and Left Eye; LE = Left Eye; RE = Right Eye; n.s. = not significant

*LCET = Tropical Endemic Limbo-Conjunctivitis

** The criteria for low and normal visual acuity according to WHO (n = 2,221 examinations) are indicated in Material and Methods.

^&^ The number of cases with the respective ocular pathology in the river basins of Kéran, Mô and Ôti are indicated in square brackets. n.s. (p>0.05): not significant

**Table 6:** Spearman’s rank correlation analyses of ocular pathologies and visual acuity of the right eye (RE) in the survey participants and significant associations (p<0.05).

RE Variable 1	RE Variable 2	Correlation Coefficient ρ	p value
Cataract	Sclerosing keratitis	0.874	< .0001
Cataract	Iridocyclitis	0.7159	0.0004
Cataract	Blindness	0.7653	0.0014
Cataract	Low vision	0.7528	0.0019
Cataract	LCET*	-0.7222	0.0035
LCET	Sclerosing keratitis	-0.7001	0.0053
Blindness	Iridocyclitis	0.8568	< .0001
Blindness	Sclerosing keratitis	0.7191	0.0038
Chorioretinitis	Sclerosing keratitis	0.6485	0.0121
Chorioretinitis	LCET	-0.6221	0.0175
Chorioretinitis	Papillitis	0.5787	0.0301
Vision Low	Sclerosing keratitis	0.8163	0.0004
Vision Low	Druzen	0.7841	0.0009
Vision Low	Iridocyclitis	0.7068	0.0047
Vision Low	Vascular retinopathy	0.6132	0.0197
Vision Low	Trichiasis	0.5822	0.0289
Vision Normal	Sclerosing keratitis	-0.7447	0.0022
Vision Normal	Iridocyclitis	-0.7415	0.0024
Druzen	Vascular retinopathy	0.7723	0.0012
Iridocyclitis	sclerosing keratitis	0.8135	0.0004

*LCET = Tropical Endemic Limbo-Conjunctivitis

## Discussion

The annual mass distribution of ivermectin during the past 25 years has greatly reduced *O*. *volvulus* infection prevalence in Togo. In the surveyed populations, the overall *O*. *volvulus* microfilarial prevalence has decreased below 5%. While this is strong evidence that elimination of onchocerciasis is a realistic outcome, in several locations in northern and central Togo, Mf-positivity ranged above 5% and 10%, respectively. The present surveys were conducted within the Ôti, Kéran and Mô river basins, in locations where patent *O*. *volvulus* infections still persisted in children aged ≤10 years and also in adults; further, progressive ocular pathology was diagnosed, and transmission of *O*. *volvulus* by *S*. *damnosum* s.l. occurred close to the studied locations.

Recently, the focus of onchocerciasis programmes has changed from control to elimination in Africa [17], and indeed, in several areas of the former OCP in Mali and Senegal [5, 18], and in foci of the Onchocerciasis Elimination Program for the Americas (OEPA) (e.g., Guatemala [7, 19] and Mexico [20]), onchocerciasis has been reported to have been eliminated. Parasite transmission has been interrupted in northern Venezuela [21] and also in western Uganda [22]. The present observations in northern and central Togo suggest that despite the long-term repeated MDA with ivermectin, elimination of onchocerciasis and interruption of parasite transmission has not yet been attained. The required duration of MDA with ivermectin for onchocerciasis elimination is under scrutiny. The ONCHOSIM and EPIONCHO models predicted that the provisional operational thresholds for treatment interruption and commencement of surveillance (pOTTIS) can be reached by annual treatments (coverage 80%) in locations with a mesoendemic Mf prevalence within 14–17 years (ONCHOSIM and EPIONCHO) [23]. The operational threshold for treatment interruption has been achieved when the Mf prevalence reaches <1.4% in the population aged ≥5 years (in this modelling comparison, children under 5 were excluded [23]). The predicted duration of MDA (coverage 80%) for hyperendemic locations was 17 years (ONCHOSIM) or >25 years (EPIONCHO). Such predictions, however, may not be applicable all throughout the endemic areas in Africa [24]. The differing parasite transmission intensities and vector species with differing abilities to transmit *O*. *volvulus* [25], the pre-control Mf prevalence [26], the persisting transmission despite long-term MDA [27, 28], and the proportion of systematic non-compliers to treatment [29] may influence the overall success in achieving the elimination goals. Modelling the elimination of river blindness using long-term data from Mali and Senegal foci has adequately shown the epidemiological trends during mass treatment; resurgence of patent *O*. *volvulus* infection, with low microfilarial prevalence, was also predicted (EPIONCHO) in areas with high pre-intervention endemicities and intense vector biting rates [30]. In Togo, the median pre-MDA Mf prevalence was at mesoendemic levels, and in some survey sites hyperendemic onchocerciasis was found with a prevalence of around 80% (Fig 1). As such, the predicted duration of MDA of 14–17 years until the pOTTIS are reached may not suffice, and in hyper-endemic locations, 25 years of MDA may not lead to elimination. It must be noted that reaching the pOTTIS is not equivalent to reaching the transmission breakpoints below which parasite populations cannot persist [23]. Current control strategies will require prolonged continuation and comprehensive operational MDA approaches which extend and account for the various factors mentioned above.

In Cameroon, and after more than 15 years of CDTI, onchocerciasis has remained mesoendemic in surveyed communities [31], and in some rainforest river basins, several communities had a microfilarial prevalence above 40% despite over a decade of CDTI [32].

In the present study in Togo, we complemented skin biopsy surveys with sensitive and specific serological, ophthalmological and entomological assessments. For the serological ELISA-based evaluations, an *O*. *volvulus* adult worm antigen extract (OvAg) and the Ov16 antigen were applied, with an all-ages sensitivity of 89% and 71%, respectively. The seroprevalence values in children and adults reflect the extent by which the endemic population is still *O*. *volvulus* positive, and further, we could distinctly identify those locations and river basins where both children and adults remain still exposed to *O*. *volvulus*. Previously, we have applied the Ov16 IgG4 ELISA as a marker of active infection in all ages with a sensitivity of 60% [33], and such an assay would miss many Mf-positive cases and underestimate the actual *O*. *volvulus* infection prevalence, notably in adult populations. *O*. *volvulus* Ov16-based ELISA is recommended for testing children aged <10 years in order to detect continuing parasite transmission, but those most exposed to *O*. *volvulus* infection are agricultural field workers at river sites, and those often are women above primary school age.

Human migration in and out of the river basins may limit treatment coverage; particularly, males aged 15 to 40 years were absent when examination and treatment were conducted (Table 1). These age groups may represent a parasite reservoir which should selectively be approached to improve therapeutic coverage with ivermectin. The reasons given by families for the absence of male members was travel and temporary work away from the villages, but the return of those absent men for agricultural activities was asserted. All surveyed villages are in close location to the Benin and Ghana borders and migration across these is common (Fig 2). Similarly, in the West Region of Cameroon, the major issue for ivermectin non-compliance was absence, firstly as a result of seasonal migration, and secondly because of fear of severe adverse events (as some areas are co-endemic with loiasis); in this area the majority of systematic non-compliers were female [34].

Our ophthalmological assessments identified ocular pathologies caused by active *O*. *volvulus* infections; the observed evolving onchocerciasis ocular lesions revealed that parasite transmission is ongoing where “river blindness” was formerly severely present. The occurrence of young individuals with punctate keratitis, with evolving iridocyclitis and chorioretinitis, indicates recent parasite exposure, whereas sclerosing keratitis and blindness in older age groups indicate prevalent cases of disease due to *O*. *volvulus* infection acquired in the past (these lesions do not respond to ivermectin treatment). In the rural communities surveyed, all-cause cataract was the main cause of visual impairment; further ocular pathologies like conjunctivitis, papillitis and non-onchocercal keratitis contribute to the low vision in the examined populations. The fewer cases of chorioretinitis, iridocylitis, punctate and sclerosing keratitis in the Ôti river basin may indicate that onchocerciasis-induced ocular pathologies have regressed, and such favourable evolution should be confirmed by larger surveys. The Ôti river basin was part of the initial OCP vector control programme since 1976, and ivermectin MDA has been applied for almost three decades. The present results support previous observations that annual ivermectin treatments eliminate and prevent the migration of *O*. *volvulus* Mf into the anterior eye chamber and cornea, with punctate keratitis resolving completely and early-stage sclerosing keratitits and iridocyclitis regressing, whereas advanced lesions of the anterior and posterior eye segment remain progressive [12, 13, 35]. Annual ivermectin treatments may prevent the emergence of ocular pathology in those populations still exposed to *O*. *volvulus* infection, but the present ophthalmological surveys support that the interruption of *O*. *volvulus* transmission for the purpose of stopping MDA has not yet been attained.

In fact, parasite transmission is ongoing in the Kéran and Mô river basins and also along the Ôti, but in the latter, the number of blackflies collected was low and time-limited; further studies are planned which will extend entomological collections over several months. The geography of the river basins in northern and central Togo is extensive; the Ôti flows from Benin crossing Togo east to west; the Kéran joins with the Koumoungou and Ôti, and the Mô starts in central Togo and continues west into the Volta river basin in Ghana. Parasite transmission has never been interrupted completely in central and northern Togo and Benin; the Ôti, Kéran and Mô river basins were SIZ where vector control and intensified ivermectin distribution needed to be continued for years after OCP closure in 2002 [9, 10]. Special interventions in the post-OCP period included continued aerial larvicide application for five additional years (2003–2007) and biannual ivermectin mass treatment (which attained a treatment coverage >80%) until the end of 2012 [9, 10]. In the Mô river basin, a high vector density was documented in 2015 and 2016, with an ABR of 15,519 bites/person/year of *S*. *damnosum* s.l. (Fig 5). This value was similar to that observed before launching the OCP [36], and this intense biting may favor parasite transmission. The positive rtPCR results confirm ongoing transmission of *O*. *volvulus*. Because whole blackflies were used (rather than just fly heads), our positive results may indicate transmission from humans to vectors as well as transmission from vectors to humans. The latter requires the presence of infective-stage larvae (L3) in the head of the vector. We have confirmation for parasite–vector contact in our analysis of body pools from northern and central regions in Togo, and in the next collections, *S*. *damnosum* s.l. head pools will be tested to gain an accurate estimate of the prevalence of flies carrying L3 larvae.

Persistent *O*. *volvulus* transmission in the study river basins can be attributed to geographical conditions which allow for trans-border migration of vectors from east to west and vice versa, notably during the rainy seasons, when *S*. *damnosum* s.l. flies may migrate across larger distances as previously observed [37, 38]. In the savannah areas of north-central Togo the savannah members of the *Simulium damnosum sensu lato* species complex prevail, notably *S*. *squamosum* in the Mô valley, *S*. *sirbanum* along the Kara and Ôti, and *S*. *soubrense* and *S*. *sanctipauli* group in eastern areas [39]. In the analysis presented here, *O*. *volvulus* DNA was detected in simuliids collected during the rainy season in the Kéran, Ôti and Mô river basins, tributaries to the Volta river basin. Thus, *O*. *volvulus* transmission control efforts should expand into cross-border collaborations [40] and ivermectin MDA should be coordinated and applied to populations that live at national frontiers, as well as be well-timed when main parasite transmission occurs.

### Conclusions

The present surveys have shown that the northern and central regions in Togo are gradually approaching the elimination of onchocerciasis [7, 8]; however, the geographical and demographic conditions in the Ôti, Kéran and Mô river basins will require continuous, comprehensively intensified and well-adapted interventions which should reach beyond the operationally standardized MDA. In formerly hyperendemic areas in northern Togo that formed part of the SIZ, biannual MDA attained >80% treatment coverage until 2015, yet many foci remain positive for onchocerciasis and parasite transmission continues. Here, the future interventional strategy may selectively adapt to the particular characteristics of the endemic populations, notably, to the seasonal migrations in and out of the river basins, to the age and gender profiles of the non-complying groups, and to the seasonal patterns of parasite transmission by the local *S*. *damnosum* s.l. vector species. Moreover, national control programmes should harmonize cross-border MDA strategies as a coordinated control measure.

## Supporting information

S1 Checklist STROBE. (DOC)

S1 Table Therapeutic coverage (median, minimum and maximum in %) by MDA of ivermectin from year 2001 to year 2015 in 32 districts in Togo, and in 4 districts in northern and central Togo (Region Savanes, Region Kara) where 11 villages were surveyed. (DOC)
