# Supplementary material for: Onchocerca volvulus infection and serological prevalence, ocular onchocerciasis and parasite transmission in northern and central Togo after decades of Simulium damnosum s.l. vector control and mass drug administration of ivermectin
Source: PLoS Negl Trop Dis. 2018 Mar 1;12(3):e0006312. doi: 10.1371/journal.pntd.0006312 (PMC5849363; doi:10.1371/journal.pntd.0006312)
Supplement: S1 Checklist — (DOC) [file pntd.0006312.s001.doc]

Manuscript PNTD-D-17-00379R2

STROBE Statement—checklist of items that should be included in reports of observational studies

|  | Item No. | Recommendation | Page  No. | Relevant text from manuscript |
| --- | --- | --- | --- | --- |
| **Title and abstract** | 1 | (*a*) Indicate the study’s design with a commonly used term in the title or the abstract | Title Page 1 | Lines 2-4  *Onchocerca volvulus* infection and serological prevalence, ocular onchocerciasis and parasite transmission in northern and central Togo after decades of *Simulium damnosum* s.l. vector control and mass drug administration of ivermectin - observational studies |
|  |  | (*b*) Provide in the abstract an informative and balanced summary of what was done and what was found | Authors Summary  Page 4 | Lines 71-86  Mass drug administration (MDA) with ivermectin has become the main tool in the efforts to control and eliminate onchocerciasis (“river blindness”). In some areas, and after many years of MDA, levels of *Onchocerca volvulus* infection (the causative parasite) have declined greatly, and elimination appears achievable. In certain river basins of northern and central Togo, the present epidemiological situation remains unknown. The guidelines of the World Health Organization recommend that before ivermectin MDA can be stopped, interruption of *O. volvulus* transmission must be demonstrated. To this end, parasitological, serological, ophthalmological, and entomological assessments were conducted in the Ôti, Kéran and Mô river basins. *O. volvulus* infections and positive antibody responses were found in children aged ≤10 years and adults. Progressive ocular onchocerciasis was diagnosed, and parasite transmission by *Simulium damnosum* s.l. (the disease vector) occurred close to the survey locations. Thus, *O. volvulus* transmission continues in northern and central Togo, and future interventions may require approaches selectively adapted to seasonal migration of non-complying endemic populations in and out of the river basins, as well as seasonal transmission by the vectors. National control programmes should harmonize cross-border MDA as a coordinated intervention. |
| Introduction | | |  | |
| Background/rationale | 2 | Explain the scientific background and rationale for the investigation being reported | Introduction  Page 5 | Lines 89-95  In large parts of West Africa, onchocerciasis has been controlled as a public health problem by the Onchocerciasis Control Programme (OCP) and the African Programme for Onchocerciasis Control (APOC) by mass drug administration (MDA) of ivermectin, and this intervention has been applied for more than two decades. In vast part of the initial control areas of the OCP, *Onchocerca volvulus* infection prevalence and intensity levels have strongly been reduced [1,2], and currently, the elimination of onchocerciasis appears achievable in certain endemic regions [3-7].  Lines 101-110  Regular epidemiological surveys conducted by the National Onchocerciasis Control Program (NOCP) have shown that after nearly three decades of MDA in most of the onchocerciasis hyper-endemic districts the *O. volvulus* microfilarial prevalence has diminished below 5% in all age groups and below 1% in children aged less than 10 years, suggesting that considerable progress has been made towards elimination of onchocerciasis according to the operational prevalence thresholds proposed in the Conceptual Framework for Elimination of Onchocerciasis by APOC [3, 8]. Parasite transmission has never been interrupted completely in central and northern Togo and Benin; the Ôti, Keran and Mô river basins were “special intervention zones” where vector control and intensified ivermectin distribution needed to be continued for years after OCP closure in 2002 [9].  Lines 114-117  Despite evidence of approaching elimination in certain regions of Togo, the current situation remains to be assessed by epidemiological, entomological and serological surveys for detection of infection in human and vector population samples according to the recent World Health Organization (WHO) guidelines [11]. |
| Objectives | 3 | State specific objectives, including any pre-specified hypotheses | Introduction  Page 7 | Lines 125-129  In the present work, parasitological, serological, ophthalmological and entomological evaluations were conducted in onchocerciasis sentinel villages in central and northern Togo to assess the current epidemiological situation and to determine whether transmission has been interrupted and ivermectin MDA can be stopped.  Our findings reveal that despite the long lasting onchocerciasis control interventions, the all-ages *O. volvulus* infection prevalence exceeded 10% in some areas, progressive ocular onchocerciasis lesions were observed in patients and parasite transmission continues in the river basins in central and northern Togo. |
| Methods | | |  | |
| Study design | 4 | Present key elements of study design early in the paper | Mat+Meth  Page 7 | Lines 151-153  Regular epidemiological surveys were conducted in Togo by the Onchocerciasis Control Programs (OCP-West Africa and NOCP-Togo) which assessed the *O. volvulus* microfilarial prevalence and intensity, the treatment coverage and compliance to the mass drug administration (MDA) with ivermectin within the program area. |
| Setting | 5 | Describe the setting, locations, and relevant dates, including periods of recruitment, exposure, follow-up, and data collection | Mat+Meth  Page 7+8 | Lines 174-186  Regular epidemiological surveys were conducted in Togo by the Onchocerciasis Control Programs (OCP-West Africa and NOCP-Togo) were performed . Such surveys were performed since 1976 during the early rainy season, and around 200 participants were recruited and examined in each selected sentinel village. All sentinel villages are located within less than 3 km of distance to rivers with known breeding sites for the blackfly vector *Simulium damnosum* sensu lato (s.l.)*.* In Togo, vector control and epidemiological surveys started in 1976 within the OCP-Phase-III-Eastern Extension in the northern river basins of Ôti, Koumoungou and Kara. In 1988, control measures and epidemiological surveys began for sentinel villages of the OCP-Southern Extension in the river basins of Mô and Mono, and at the same time, also in southern Togo in the river basins of Amou, Anie and Mono. The total number of sentinel villages in Togo included in the epidemiological surveys was 363, and the endemic populations were repeatedly examined over time.  The present parasitological, serological and ophthalmologic surveys were performed in the central and northern regions of Togo (Régions Savanes and Kara). In these regions, 11 villages were selected by the National Onchocerciasis Control Program (NOCP) for annual survey. Figure 1 shows the selected villages and their location in Togo. Three villages are located in the Région Savanes in the river basin of Ôti, i.e. Pancérys, Boutchakou and Koukoumbou. Four villages within the Region Kara are situated along the river Kara, i.e. Goulbi, Tchitchira, Koukoumbou Solla and Kpantiiyagou. Further four villages from the Région Kara are located in the river basin of Mô, i.e. Bawlesi, Mô-Village, Katcha-Konkomba and Saboundi. All sentinel villages are located within less than 1 km distance to the rivers Ôti, Karan or Mô with known breeding sites for the black fly vector *Simulium spp..* |
| Participants | 6 | (*a*) *Cohort study*—Give the eligibility criteria, and the sources and methods of selection of participants. Describe methods of follow-up  *Case-control study*—Give the eligibility criteria, and the sources and methods of case ascertainment and control selection. Give the rationale for the choice of cases and controls  *Cross-sectional study*—Give the eligibility criteria, and the sources and methods of selection of participants | Mat+Meth  Pages  9-10 | Lines 185-189  Before ivermectin MDA (delivered by community-directed drug distributors, CDDs), participants gave their informed consent for the collection of skin biopsies to detect *O. volvulus* microfilariae (Mf). Participation and examination were conducted by family and followed the status: heads of family (parents), children, brothers, uncles, aunts, and grandparents.  Lines 213-223  The ophthalmology examinations were performed by MB and TS and ocular pathologies, their grades of evolution and extent were classified as described previously [12]. All participants acknowledged having received ivermectin annually for several years through community directed treatment with ivermectin (CDTI). In the surveyed villages, therapeutic ivermectin coverage of the eligible population had been ≥80% during the past 10 years. The anterior eye segment was examined by slit lamp (Haag Streit 900) after participants were asked to sit with their heads bent between their knees for at least two minutes. This position promotes the migration of microfilariae within the anterior chamber of the eye (MFAC) to be seen and counted. The examination of the posterior segment was done with an ophthalmoscope after pupil dilation with 1% tropicamide and 10% epinephrine hydrochloride. |
|  |  | (*b*)*Cohort study*—For matched studies, give matching criteria and number of exposed and unexposed  *Case-control study*—For matched studies, give matching criteria and the number of controls per case. |  | Not applicable |
| Variables | 7 | Clearly define all outcomes, exposures, predictors, potential confounders, and effect modifiers. Give diagnostic criteria, if applicable | Mat+Meth  Pages  9-12 | Lines 184-235:  Skin biopsy collection and examinations  From each participant, a skin biopsy was taken from each the left and right iliac crest (for a total of two snips) with a sterile 2-mm Holth corneo-scleral punch biopsy tool. Immediately, skin snips were placed on glass slides and incubated with physiological saline solution for 30 minutes. Each biopsy was microscopically examined for emerging *O. volvulus* Mf and their number counted. Blood sample collection  Blood drops were collected from fingertips pricked with a sterile lancet on Whatman 903 Protein Saver Cards. The cards were air dried, sealed in plastic bags and stored at 4°C until further use. As per skin biopsies and for each participant, village of residence, family affiliation, age, sex, number of ivermectin treatment rounds received and microfilarial counts in skin biopsies were recorded.  Ophthalmological examinations  The ophthalmology examinations were performed by MB and TS and ocular pathologies, their grades of evolution and extent were classified as described previously.  Next to individual data (age, sex, occupation, village, number of ivermectin treatments), the microfilarial load in the anterior eye segment, punctate and sclerosing keratitis and iridocyclitis were recorded. Onchocerciasis cases with punctate keratitis were grouped according to the presence of dead Mf in the cornea (DMFC) or living Mf in the anterior chamber (MFAC) and further classified as low (presence of 1-10 Mf), moderate (11-20 Mf) or high (>20 Mf). Ocular lesions of the posterior segment were coded as evolving or advanced according to the classification adopted by WHO/OCP [13]. The ocular examinations included the testing of visual acuity eye by eye with an illiterate E chart (SNELEN) placed 6 meters away from the patient’s seat, and visual acuity was graded according to WHO/OCP criteria  Lines 237-257: Serological tests  For the OvAg-IgG4 ELISA, an adult worm antigen extract from male and female *Onchocerca volvulus* was used [14,15]; for the Ov16-IgG4 ELISA, the recombinant *O. volvulus*-specific antigen Ov16 was applied to measure serological IgG4 responses.  Lines 259-271: Collection of vector black flies *Simulium damnosum s.l.*  The collection of *S. damnosum s.l.*  was conducted at specific catch points at river sites by trained fly catchers in proximity to sentinel villages in the river basin of Ôti (village Pancerys/Region Savanes), Kara (village Tchitchira/Region Kara), Mô (village Baghan/Region Kara and Bouzalo/Region Central) during the rainy season on five successive days in late August and beginning September 2015. In addition, repeated weekly collections of *S. damnosum s.l.*  were continued at river site Mô in proximity to the village Bouzalo (Region Centrale) from September 2015 until August 2016. The sampling procedure was the same as above, and this long-term 2015-2016 collection was to determine the annual biting rate (ABR). |
| Data sources/measurement | 8* | For each variable of interest, give sources of data and details of methods of assessment (measurement). Describe comparability of assessment methods if there is more than one group | Page 15 | Parasitological, ophthalmologic and serological examinations were completed with 1,455 participants from 11 onchocerciasis sentinel villages, and the *O. volvulus* transmission by Simulium spp. was evaluated.  The 11 villages were selected by the National Onchocerciasis Control Program (NOCP) for annual survey. Three villages are located in the Région Savanes in the river basin of Ôti, i.e. Pancérys, Boutchakou and Koukoumbou. Four villages within the Region Kara are situated along the river Kara, i.e. Goulbi, Tchitchira, Koukoumbou Solla and Kpantiiyagou. Further four villages from the Région Kara are located in the river basin of Mô, i.e. Bawlesi, Mô-Village, Katcha-Konkomba and Saboundi. All sentinel villages are located within less than 1 km distance to the rivers Ôti, Karan or Mô with known breeding sites for the black fly vector *Simulium spp..*  Regular epidemiological surveys conducted by the OCP and NOCP, and around 200 participants were recruited and examined in the selected sentinel village. All sentinel villages are located within less than 3 km distance to rivers with known breeding sites for the black fly vector *Simulium damnosum s.l..* |
| Bias | 9 | Describe any efforts to address potential sources of bias |  | Was not addressed. |
| Study size | 10 | Explain how the study size was arrived at | Page 15 | For the present observations examinations were completed with 1,455 participants from 11 onchocerciasis sentinel villages according to the mentioned criteria, and around 150 participants were recruited and examined in the selected sentinel village. A total of 1,455 study participants from 11 NOCP (National Onchocerciasis Control Programme) sentinel villages volunteered and gave their informed consent for participation. In the river basins of Ôti 22.3% (n=324) of the participants were recruited, from the river basins of Kéran originated 37.0% (n=539) and 40.7% (n=592) were village residents in the river basin of Mô. |
| Continued on next pageQuantitative variables | 11 | Explain how quantitative variables were handled in the analyses. If applicable, describe which groupings were chosen and why | Pages 10-12 | Assessed were the O. volvulus microfilarial prevalence and intensity. From each participant, the village of resident, age, sex, and microfilarial counts in skin biopsies were recorded.  With ophthalmology age, sex, village, the microfilarial load in the anterior eye segment, punctate and sclerosing keratitis and iridocyclitis were recorded. Onchocerciasis cases with punctate keratitis were grouped according to the presence of dead microfilariae (MF) in the cornea (DMFC) or living MF in the anterior chamber (MFAC) and further classified as low (presence of 1-10 MF), average (between 11-20 MF) or high (>20 MF). Ocular lesions of the posterior segment were coded as evolving or advanced according to the classification adopted by WHO/OCP [13]. The ocular examinations included the testing of visual acuity eye by eye with an illiterate E chart (SNELEN) placed 6 meters away from the patient’s seat, and visual acuity was graded according to WHO/OCP criteria.  Serological parameter (parasite-specific IgG4 responses) were optical densities (OD) as determined by ELISA. |
| Statistical methods | 12 | (*a*) Describe all statistical methods, including those used to control for confounding | Pages 13-14 | Data were entered in Microsoft Excel and analyses were conducted with the statistical software SAS JMP 11.1.1. For Mf prevalence values, the 95% confidence intervals (95% CI, normal approximation to the binomial distribution) were calculated. The sensitivity of the *O. volvulus*-specific IgG4 ELISA was determined with a contingency analysis. For explorative data analyses, the two-sample Wilcoxon test was applied to evaluate differences between groups. The Chi-square test was used to test differences between examined males and females (e.g. participation rates). Fisher’s exact test (two-sided) was applied to compare Mf-prevalence and the ELISA IgG4-OvAg and Ov-16 positive responses between river basins (Kéran, Ôti, Mô), and the number of Ov-150 DNA positive *Simulium damnosum* s.l. pools from Ôti/Pancery, Kéran/Baghan, Kéran/Tchitichira and Mô/Bouzalo. One-sided Fisher's exact test was used to evaluate differences in the prevalence of ocular pathologies in patients from the Ôti, Kéran and Mô river basins. Correlations between ophthalmological variables as well as between these and age were explored with Spearman correlation coefficient. For multiple testing, the application of the Bonferroni Holm adjustment (11 villages, 3 river basins, 7 age groups, microfilarial prevalences, IgG4 responses) resulted in an alpha level of 0.0023. For multiple comparisons, and to avoid type I errors, differences between groups were analyzed by the Tukey-Kramer Test. |
|  |  | (*b*) Describe any methods used to examine subgroups and interactions | Page 10-14 | Assessed were the O. volvulus microfilarial prevalence and intensity. From each participant, the village of resident, age, sex, and microfilarial counts in skin biopsies were recorded.  With ophthalmology age, sex, village, the microfilarial load in the anterior eye segment, punctate and sclerosing keratitis and iridocyclitis were recorded. Onchocerciasis cases with punctate keratitis were grouped according to the presence of dead microfilariae (MF) in the cornea (DMFC) or living MF in the anterior chamber (MFAC) and further classified as low (presence of 1-10 MF), average (between 11-20 MF) or high (>20 MF). Ocular lesions of the posterior segment were coded as evolving or advanced according to the classification adopted by WHO/OCP [13].  The ocular examinations included the testing of visual acuity eye by eye with an illiterate E chart (SNELEN) placed 6 meters away from the patient’s seat, and visual acuity was graded according to WHO/OCP criteria.  Serological parameter (parasite-specific IgG4 responses) were optical densities (OD) as determined by ELISA, and *Onchocerca volvulus* microfilariae (Mf) prevalence in age groups of examined participants from NOCP sentinels villages in central and northern Togo. |
|  |  | (*c*) Explain how missing data were addressed |  | From n=41 participants age is missing. |
|  |  | (*d*) *Cohort study*—If applicable, explain how loss to follow-up was addressed  *Case-control study*—If applicable, explain how matching of cases and controls was addressed  *Cross-sectional study*—If applicable, describe analytical methods taking account of sampling strategy |  | Not applicable. |
|  |  | (*e*) Describe any sensitivity analyses | Page 18  Page 30-31 | The sensitivity of the IgG4 ELISAs based on *Onchocerca volvulus* adult worm antigen (OvAg) and of the *O. volvulus*-specific recombinant antigen Ov16 (right panel) for the detection of patent *O.volvulus* infection (microfilaria (Mf) positive) was evaluated. The sensitivities of the OvAg- and Ov16-specific IgG4-ELISAs were 89.2% and 71.4% to detect microfilariae (Mf)-positive participants, and IgG4 response negative to OvAg and Ov16 were 10.8% and 28.4% of the Mf-positive participants, respectively.  In the present study in Togo, we complemented sensitive and specific serological, ophthalmological and entomological assessments. For the serological ELISA-based evaluations, an *O. volvulus* adult worm antigen extract (OvAg) and the Ov16 antigen were applied, with an all-ages sensitivity of 89% and 71%, respectively. The seroprevalence values in children and adults reflect the extent by which the endemic population is still *O. volvulus* positive, and further, we could distinctly identify those locations and river basins where both children and adults remain still exposed to *O. volvulus*. Previously, we have applied the Ov16 IgG4 ELISA as a marker of active infection in all ages with a sensitivity of 60% [33], and such an assay would miss many Mf-positive cases and underestimate the actual *O. volvulus* infection prevalence, notably in adult populations. *O. volvulus* Ov16-based ELISA is recommended for testing children aged <10 years in order to detect continuing parasite transmission, but those most exposed to *O. volvulus* infection are agricultural field workers at river sites, and those often are women above primary school age. |
| Results | | | | |
| Participants | 13* | (a) Report numbers of individuals at each stage of study—eg numbers potentially eligible, examined for eligibility, confirmed eligible, included in the study, completing follow-up, and analysed | Page 14-26 | The *O. volvulus* microfilarial prevalence in onchocerciasis sentinel villages located in the major river basins of Ôti, Kéran, Kara, Mô, Koumoungou, Anie and Mono declined markedly (Fig. 1), and until the year 2014, the median prevalence of *O. volvulus* infections dropped below 5%, but in several locations the Mf-positivity exceeded this level in the river basins of Ôti, Kéran and Mô.  A total of 1,455 study participants from 11 NOCP (National Onchocerciasis Control Programme) sentinel villages volunteered and gave their informed consent for participation. In the river basins of Ôti 22.3% (n=324) of the participants were recruited, from the river basins of Kéran originated 37.0% (n=539) and 40.7% (n=592) were village residents in the river basin of Mô (Table 1).  Information on age is missing from 41 of the 1,455 participants, so data in Table 1 are reported for a total 1,414 individuals. Of these, 819 were females and 595 males. The median age in females and males was 30 and 29 years, respectively. Until the age of 15 years, girls and boys were similarly represented in the survey, but participation in examination (and treatment) of men aged 16 to 40 years decreased significantly (Table 1). The Chi-square test was applied to compare differences between female and male survey participation within age groups, indicating a statistically significant difference with greater participation of females (16-20y: p=0.0007; age groups 21-25y, 26-30y and 31-35y: for each p<0.0001; 36-40y: p=0.04). In the age groups above 40 years, differences in participation between the sexes were not significant.  The main ocular pathologies in the examined village populations, reported for the right eye, were papillitis (19.5%), cataract (17.6%), chorioretinitis (9.8%), conjunctivitis (7.8%), tropical limbo-cojunctivitis (LCET) (6.3%), iridocyclitis (4.6%), sclerosing keratitis (3.9%) and blindness of either eye (7.4%) (Table 5). Of note were punctate keratitis lesions with 1-10 Mf of *O. volvulus* in the cornea present in children (aged 12-15 years) and adults (28-52 years) and sclerosing keratitis in adults (Table 5, Fig 5). In four cases, alive or dead *O. volvulus* Mf were detected in the anterior chamber of the eye. Iridocyclitis in evolution (n=7) was found in youngsters and adults; retinal lesions (chorioretinis) were present in younger adults and older ages (Fig 5) with n=19 being in evolution and n=41 at an advanced stage. Evolving cataract (all causes) was diagnosed in a few children and mainly in older ages. Cataracts caused by *O. volvulus* infection and blindness caused by onchocerciasis were observed in individuals aged above 50 years (Fig 5). |
|  |  | (b) Give reasons for non-participation at each stage | Page 29 | Human migration in and out of the river basins may limit treatment coverage; particularly, males aged 15 to 40 years were absent when examination and treatment were conducted (Table 1). These age groups may represent a parasite reservoir which should selectively be approached to improve therapeutic coverage with ivermectin. The reasons given by families for the absence of male members was travel and temporary work away from the villages, but the return of those absent men for agricultural activities was asserted. All surveyed villages are in close location to the Benin and Ghana borders and migration across these is common (Fig 2). |
|  |  | (c) Consider use of a flow diagram |  |  |
| Descriptive data | 14* | (a) Give characteristics of study participants (eg demographic, clinical, social) and information on exposures and potential confounders | Page 9  Page 30 | The present observations and examinations were completed with 1,455 participants from 11 onchocerciasis sentinel villages according to the mentioned criteria, and around 150 participants were recruited and examined in the selected sentinel village. Participants from 11 NOCP (National Onchocerciasis Control Programme) sentinel villages volunteered and gave their informed consent for participation. In the river basins of Ôti 22.3% (n=324) of the participants were recruited, from the river basins of Kéran originated 37.0% (n=539) and 40.7% (n=592) were village residents in the river basin of MôAround 200 participants were recruited and examined in the selected sentinel village. All sentinel villages are located within less than 3 km distance to rivers with known breeding sites for the black fly vector *Simulium damnosum s.l..*  Parasite transmission is ongoing in the Kéran and Mô river basins and also along the Ôti, but in the latter, the number of blackflies collected was low and time-limited; further studies are planned which will extend entomological collections over several months. The positive rtPCR results confirm ongoing transmission of *O. volvulus*. Because whole blackflies were used (rather than just fly heads), our positive results may indicate transmission from humans to vectors as well as transmission from vectors to humans. The latter requires the presence of infective-stage larvae (L3) in the head of the vector. We have confirmation for parasite–vector contact in our analysis of body pools from northern and central regions in Togo, and in the next collections, *S. damnosum* s.l. head pools will be tested to gain an accurate estimate of the prevalence of flies carrying L3 larvae. |
|  |  | (b) Indicate number of participants with missing data for each variable of interest |  | From n=41 age is missing. |
|  |  | (c) *Cohort study*—Summarise follow-up time (eg, average and total amount) |  | Not addressed |
| Outcome data | 15* | *Cohort study*—Report numbers of outcome events or summary measures over time | Page 18  Page 14  Page 18 | The overall Mf prevalence in the survey participants was 5.7%. In the river basins of Ôti, Kara and Mô, the Mf prevalence ranged from 0.8-5.4%, 7.7-13.6% and 0-8.6%, respectively (Table 2). Among children of 1 to 10 years, the Mf prevalence was 2.3% and in the adult age groups (>18years) the Mf prevalence ranged from 3.7-10.8%.  In 1976, the prevalence of *O. volvulus* infection exceeded in most locations 50%, and 20 years later, Mf-positivity in the survey populations decreased to below 20% (median) (Fig 1). The *O. volvulus* microfilarial prevalence in onchocerciasis sentinel villages located in the major river basins of Ôti, Keran, Kara, Mô, Koumoungou, Anie and Mono declined markedly (Fig 1), and until the year 2014, the median prevalence of *O. volvulus* infections dropped below 5%, but in several locations the Mf-positivity exceeded this level and ranged up to 15% in the river basins of Ôti, Keran and Mô.  The all-ages positive IgG4 serologic reactivity to the OvAg was 60.5% (880/1,455; Table 2). The observed serological responsiveness heightens continuously with increasing age (Fig. 3) and attained a maximum level around the age of 50 years. During the first two decades of age the mean OvAg-specific IgG4 reactivity remained low in most cases, then from 16 years onwards an enhanced responsiveness was observed, and from 20 years of age and older the mean participants' serologic IgG4 responses to OvAg continued steadily to rise until the fifth decade of age (Fig. 3). In children of ≤10 years, 29.1% showed a positive IgG4 serological response to OvAg (n=172, Table 2) and in some villages positive IgG4 responses were above 40%. Of note, in the river basins of Keran, Mô and Ôti, children responded IgG4-positive to the *O. volvulus* antigen (Table 2) with 51.7%, 23.5% and 12.7%, respectively. |
|  |  | *Case-control study—*Report numbers in each exposure category, or summary measures of exposure | Page 32-33 | Parasite transmission and thus exposure to infection is ongoing in the river basins of the Keran and Mô rivers and supposedly also along Ôti. Eight out of 35 pools from Tchitchira were positive for Ov150-DNA (22.8%) and the calculated prevalence of *O. volvulus* in *Simulium spp.* black flies was 1% [13].  In Baghan, three positive pools were detected (2.9%) and the calculated parasite prevalence was 0.1%.  In Mô, five positive *Simulium spp.* pools were identified (11.3%) with a 0.5% prevalence of *O. volvulus* in *Simulium spp.* black flies (Table 4).  At Pancerys in the river basin of the Ôti two *O. volvulus*-positive *Simulium spp.* pools were found, and the calculated parasite prevalence was 0.2%. .  In the river basin of Mô a high vector density was detected in 2015 and 2016 with an annual biting rate (ABR) of n=15,519 *S. damnosum s.l.* (Fig 4), being similarly high as observed before launching OCP [35], and this may favor parasite transmission. The positive rtPCR results confirm ongoing transmission of *O. volvulus*. Because whole blackflies were used (rather than just fly heads), our positive results may indicate transmission from humans to vectors as well as transmission from vectors to humans. The latter requires the presence of infective-stage larvae (L3) in the head of the vector. We have confirmation for parasite–vector contact in our analysis of body pools from northern and central regions in Togo, and in the next collections, *S. damnosum* s.l. head pools will be tested to gain an accurate estimate of the prevalence of flies carrying L3 larvae.  Persistent *O. volvulus* transmission in the study river basins can be attributed to geographical conditions which allow for trans-border migration of vectors from east to west and vice versa, notably during the rainy seasons, when *S. damnosum* s.l. flies may migrate across larger distances as previously observed. |
|  |  | *Cross-sectional study—*Report numbers of outcome events or summary measures |  |  |
| Main results | 16 | (*a*) Give unadjusted estimates and, if applicable, confounder-adjusted estimates and their precision (eg, 95% confidence interval). Make clear which confounders were adjusted for and why they were included | Page 18  Page 14  Page 18 | The overall Mf prevalence in the survey participants was 5.7%. In the river basins of Ôti, Kara and Mô, the Mf prevalence ranged from 0.8-5.4%, 7.7-13.6% and 0-8.6%, respectively (Table 1). Among children of 1 to 10 years, the Mf prevalence was 2.3% and in the adult age groups (>18years) the Mf prevalence ranged from 3.7-10.8% (Table 1).  When OCP measures started in 1976, the prevalence of *O. volvulus* infection exceeded in most locations 50%, and 20 years later, Mf-positivity in the survey populations decreased to below 20% (median) (Fig 1). The *O. volvulus* microfilarial prevalence in onchocerciasis sentinel villages located in the major river basins of Ôti, Keran, Kara, Mô, Koumoungou, Anie and Mono declined markedly (Fig 1), and until the year 2014, the median prevalence of *O. volvulus* infections dropped below 5%, but in several locations the Mf-positivity exceeded this level and ranged up to 15% in the river basins of Ôti, Keran and Mô.  The all-ages positive IgG4 serologic reactivity to the OvAg was 60.5% (880/1,455; Table 2). The observed serological responsiveness heightens continuously with increasing age (Fig 3) and attained a maximum level around the age of 50 years. During the first two decades of age the mean OvAg-specific IgG4 reactivity remained low in most cases, then from 16 years onwards an enhanced responsiveness was observed, and from 20 years of age and older the mean participants' serologic IgG4 responses to OvAg continued steadily to rise until the fifth decade of age (Fig. 3). In children of ≤10 years, 29.1% showed a positive IgG4 serological response to OvAg (n=172, Table 2, Fig 3) and in some villages positive IgG4 responses were above 40%. Of note, in the river basins of Keran, Mô and Ôti, children responded IgG4-positive to the *O. volvulus* antigen (Table 2) with 51.7%, 23.5% and 12.7%, respectively. |
|  |  | (*b*) Report category boundaries when continuous variables were categorized |  |  |
|  |  | (*c*) If relevant, consider translating estimates of relative risk into absolute risk for a meaningful time period |  |  |

Continued on next page

| Other analyses | 17 | Report other analyses done—eg analyses of subgroups and interactions, and sensitivity analyses |  |  |
| --- | --- | --- | --- | --- |
| Discussion | | | | |
| Key results | 18 | Summarise key results with reference to study objectives | Page 27 | The annual mass distribution of ivermectin during the past 25 years has greatly reduced *O. volvulus* infection prevalence in Togo. In the surveyed populations, the overall *O. volvulus* microfilarial prevalence has decreased below 5%. While this is strong evidence that elimination of onchocerciasis is a realistic outcome, in several locations in northern and central Togo, Mf-positivity ranged above 5% and 10%, respectively. The present surveys were conducted within the Ôti, Kéran and Mô river basins, in locations where patent *O. volvulus* infections still persisted in children aged ≤10 years and also in adults; further, progressive ocular pathology was diagnosed, and transmission of *O. volvulus* by *S. damnosum* s.l. occurred close to the studied locations. |
| Limitations | 19 | Discuss limitations of the study, taking into account sources of potential bias or imprecision. Discuss both direction and magnitude of any potential bias | Page 29-34 | Human migration in and out of the river basins may limit treatment coverage, particularly males aged from around 20 to 45 years were absent when treatment and re-examination were conducted (Table 1). These age groups may represent a parasite reservoir which selectively should be approached to improve therapeutic coverage with ivermectin.  In the present surveys in Togo we complemented the skin biopsy procedure with sensitive and specific serologic and ophthalmologic examinations. For the serological ELISA-based evaluations an *O. volvulus* adult worm antigen extract and the Ov16 antigen ELISA were applied. This allowed us to detect in children and in adults the infection prevalence which reflects the extent by which the endemic population is still *O. volvulus* positive, and further, we could distinctly identify those locations and river basins where both children and adults remain constantly exposed to *O. volvulus*.  Our ophthalmologic assessment identified ocular pathologies caused by active *O. volvulus* infections; the observed evolving onchocerciasis ocular lesions disclosed that parasite transmission is ongoing where “river blindness” was formerly severely present. The patients of younger age with keratitis punctata, with evolving iridocyclitis and chorioretinitis are indices for recent parasite exposure, while onchocerciasis-caused sclerosing keratitis and blindness were present in older age groups suggesting persistent *O. volvulus* infections. In the rural communities surveyed, cataract was the main cause for visual impairment being present in all ages, and further ocular pathologies like conjunctivitis, papillitis and non-onchocercal keratitis have to be considered as contributors to the low vision in the examined populations. Regular annual ivermectin treatments will eliminate and prevent the migration of *O. volvulus* microfilariae into the anterior eye chamber and cornea; keratitis punctata lesions will resolve completely and early-stage sclerosing keratitits and iridocyclitis regress, whilst advanced lesions of the anterior and posterior eye segment will remain progressive [12, 13, 40]. |
| Interpretation | 20 | Give a cautious overall interpretation of results considering objectives, limitations, multiplicity of analyses, results from similar studies, and other relevant evidence | Page 29-34 | Parasitological, clinical, serological and entomological evaluations were conducted in onchocerciasis sentinel villages in central and northern Togo. Our findings reveal that despite the long lasting onchocerciasis control interventions, the all-ages *O. volvulus* infection prevalence exceeded 10% in some areas, progressive ocular onchocerciasis lesions were observed in patients and parasite transmission continues in the river basins in central and northern Togo.  The reasons given by families for the non-compliance to MDA of male members was travel and temporary work away from the village, however, those absent men were asserted to return back for agricultural activities. All surveyed villages are in close location to the Benin and Ghana borders and migration across these is common. Similarly in the West Region of Cameroon, the major issues for ivermectin non-compliance was absence, often as a result of seasonal migration, second, the fear of severe adverse effects and the majority of systemic non-compliers were female [34]. Also in Cameroon, after more than 15 years of community-directed treatments with ivermectin (CDTI), onchocerciasis remained meso-endemic in selectively surveyed communities [31], and in some rain forest river basins several communities had a microfilarial prevalence above 40% despite over a decade of CDTI [32]. |
| Generalisability | 21 | Discuss the generalisability (external validity) of the study results | Page 34 | The present surveys have shown that the northern and central regions in Togo are progressively approaching to the elimination of onchocerciasis [7, 8], however, the geographic and demographic conditions in the river basins of Ôti, Keran and Mô will require continuous, comprehensively intensified and well-adapted interventions which should reach beyond the operationally standardized MDA. In formerly hyperendemic areas in northern Togo that formed part of the special intervention zones, biannual MDA attained >80% treatment coverage until 2015, yet many foci remain positive for onchocerciasis and parasite transmission continues.  Here, the future interventional strategy may selectively adapt to the particular characteristics of the endemic populations, notably, to the seasonal migrations in and out of the river basins, to the profiles of the non-complying groups, to the seasonally spiking parasite transmission, and national control programs should approach to harmonize cross-border MDA as a coordinated control measure. |
| Other information | |  | | |
| Funding | 22 | Give the source of funding and the role of the funders for the present study and, if applicable, for the original study on which the present article is based |  | This work was supported by the Commission of the European Community FP7 Project (Grant Acronym E-PIAF #242131), the research program of the Bundesministerum für Bildung und Forschung (BMBF grant 01KA1008) and the Togolese Ministry of Health (Authorisation 338/2015/MSPS/CAB/SG). For support we thank the laboratory and  technical staff from the Centre Hospitalier Regional (CHR) de Sokodé/Togo and from the National Program for Onchocerciasis Control (NOCP), Kara/Togo. The funders had no role in study design, data collection and analysis, decision to publish, or preparation of the manuscript. |

*Give information separately for cases and controls in case-control studies and, if applicable, for exposed and unexposed groups in cohort and cross-sectional studies.

**Note:** An Explanation and Elaboration article discusses each checklist item and gives methodological background and published examples of transparent reporting. The STROBE checklist is best used in conjunction with this article (freely available on the Web sites of PLoS Medicine at http://www.plosmedicine.org/, Annals of Internal Medicine at http://www.annals.org/, and Epidemiology at http://www.epidem.com/). Information on the STROBE Initiative is available at www.strobe-statement.org.
