# Supplementary material for: Onchocerca volvulus infection and serological prevalence, ocular onchocerciasis and parasite transmission in northern and central Togo after decades of Simulium damnosum s.l. vector control and mass drug administration of ivermectin
Source: PLoS Negl Trop Dis. 2018 Mar 1;12(3):e0006312. doi: 10.1371/journal.pntd.0006312 (PMC5849363; doi:10.1371/journal.pntd.0006312)
Supplement: S1 Table — (DOC) [file pntd.0006312.s002.doc]

Supporting Information

S1 Table

Therapeutic coverage (median, minimum and maximum in %) by MDA of ivermectin from year 2001 to year 2015 in 32 districts in Togo, in 4 districts in northern and central Togo (Region Savanes, Region Kara) where 11 villages were surveyed

| Therapeutic coverage (in %) by MDA of ivermectin from year 2001 to year 2015 in 32 districts in Togo | | | | Therapeutic coverage (in %) by MDA of ivermectin from year 2001 to year 2015 in 4 districts in Togo where 11 villages were surveyed | | |
| --- | --- | --- | --- | --- | --- | --- |
| Year | median (%) | minimum (%) | maximum (%) | median (%) | minimum (%) | maximum (%) |
| 2001 | 74,5 | 64 | 82 | 74 | 71 | 80 |
| 2002 | 76,5 | 69 | 82 | 72 | 69 | 80 |
| 2003 | 80 | 63 | 89 | 84 | 78 | 87 |
| 2004 | 85 | 80 | 89 | 84 | 82 | 86 |
| 2005 | 85 | 81 | 88 | 85 | 84 | 87 |
| 2006 | 85 | 78 | 89 | 85 | 85 | 86 |
| 2007 | 85 | 78 | 89 | 83,5 | 78 | 85 |
| 2008 | 85,5 | 84 | 88 | 85 | 85 | 86 |
| 2009 | 86 | 84 | 88 | 85 | 84 | 86 |
| 2010 | 85,5 | 80 | 88 | 82,5 | 80 | 85 |
| 2011 | 83,5 | 76 | 89 | 83,5 | 77 | 85 |
| 2012 | 83,5 | 53 | 89 | 84,5 | 81 | 86 |
| 2013 | 84 | 80 | 89 | 83 | 81 | 85 |
| 2014 | 82 | 68 | 89 | 82 | 81 | 85 |
| 2015 | 83 | 69 | 86 | 80 | 78 | 83 |
